# miR-204-5p is sponged by TUG1 to aggravate neuron damage induced by focal cerebral ischemia and reperfusion injury through upregulating COX2

**DOI:** 10.1038/s41420-022-00885-x

**Published:** 2022-02-28

**Authors:** Pu Xiang, Jian Hu, Hong Wang, Ying Luo, Chao Gu, Xiaodan Tan, Yujun Tu, Wenjia Guo, Lin Chen, Lin Gao, Rongchun Chen, Junqing Yang

**Affiliations:** 1 Department of Pharmacology, Chongqing Medical University, the Key Laboratory of Biochemistry and Molecular Pharmacology, Chongqing, 400016 China; 2 Department of Pharmacy, Dianjiang People’s Hospital of Chongqing, Chongqing, 408300 China; 3 Department of Hepatobiliary Surgery, Dianjiang People’s Hospital of Chongqing, Chongqing, 408300 China; 4 Department of Neurology, Dianjiang People’s Hospital of Chongqing, Chongqing, 408300 China

**Keywords:** Cell death in the nervous system, Stroke

## Abstract

Studies have reported that miR-204-5p is involved in multiple biological processes. However, little is known about the expression and mechanism of miR-204-5p in cerebral ischemia and reperfusion injury. This study found that miR-204-5p expression was significantly downregulated in the blood of patients with ischemic stroke, MCAO/R rat brains, and OGD/R neurons. Overexpression of miR-204-5p markedly reduced infarct volume and neurological impairment and alleviated the inflammatory response in vivo. miR-204-5p promoted neuronal viability and reduced apoptotic cells in vitro. Mechanically, miR-204-5p was negatively regulated by the expression lncRNA TUG1 upstream and down-regulated COX2 expression downstream. Therefore, the TUG1/miR-204-5p/COX2 axis was involved in ischemia and reperfusion-induced neuronal damage. This finding may provide a novel strategy for the treatment of cerebral ischemia and reperfusion injury.

## Introduction

Reperfusion therapies are the primary treatment principle for ischemic stroke (IS) [1–3]. However, reperfusion after cerebral ischemia can aggravate the pathological damage of ischemic brain tissue, causing cerebral ischemia and reperfusion injury (CIRI) [4]. Reperfusion strongly promotes inflammation development and increases the blood-brain barrier permeability [5]. Cyclooxygenase (COX) is the rate-limiting enzyme in arachidonic acid metabolism. Specifically, COX2 is critically involved in the loss of cortical and striatal neurons [6]. COX2 is strongly expressed in autopsy samples from stroke patients and CIRI rats [7–11]. Neurological function improved significantly in COX2-deficient mice but was exacerbated in COX2-overexpression mice [12, 13]. Although selective COX2 inhibitors can reduce CIRI to some extent [14–18], adverse drug events can occur. It is necessary to explore newer and safer endogenous drugs to improve CIRI by regulating COX2.

microRNAs (miRNAs) are key regulatory molecules for gene expression related to CIRI physiological and pathological processes [19]. As a tumor suppressor, miR-204-5p has reduced expression in tumors [20]. Long-chain noncoding RNAs (lncRNAs) can serve as molecular sponges of miRNAs. The taurine upregulated gene 1 (TUG1) is closely related to human diseases [21–24]. TUG1 sponged microRNA-9 and promoted neuronal apoptosis by upregulating Bcl2l11 under ischemia [25]. These studies suggest that TUG1 may be involved in the IS pathological processes. In calcification of the aortic valve, TUG1 bound directly to miR-204-5p and upregulated the target gene Runx2 of miR-204-5p to promote osteoblast differentiation [26].

Little is known about the expression and mechanism of miR-204-5p in CIRI and how TUG1 acts with miR-204-5p at the molecular level. We hypothesize that the TUG1/miR-204-5p/COX2 axis is involved in CIRI. Our present study aimed to investigate the possible mechanisms of TUG1/miR-204-5p/COX2 axis in CIRI. The results may provide insights on CIRI and offer a potential approach to improve IS treatment.

## Results

### TUG1, miR-204-5p and COX2 mRNA were involved in the IS development

**Fig. 1:**
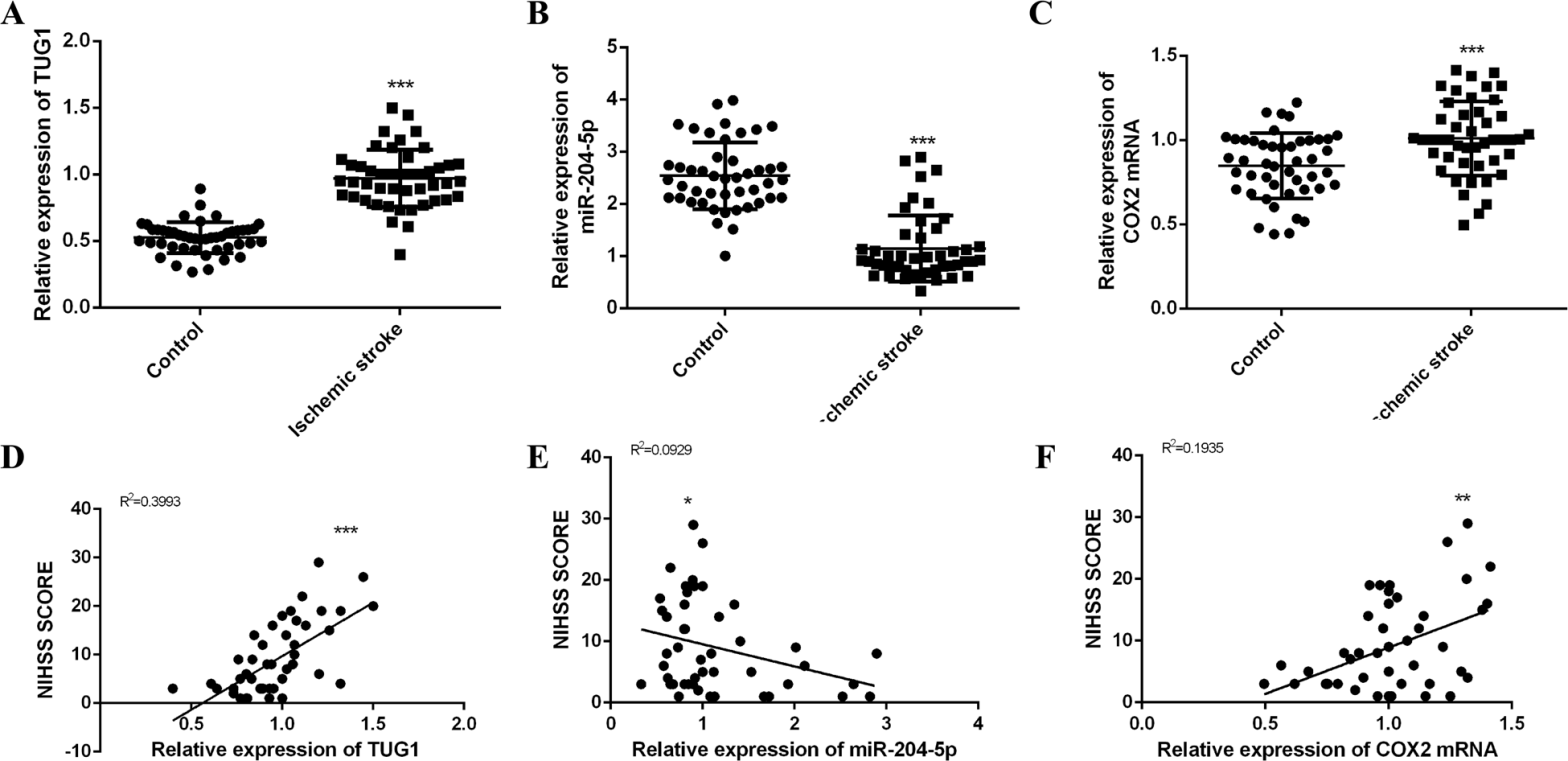
TUG1, miR-204-5p and COX2 mRNA were involved in the development of ischemic stroke. Expressions of TUG1 (**A**), miR-204-5p (**B**) and COX2 mRNA (**C**) in blood of the subjects detected by qRT-PCR. Linear regression analysis was conducted to each patient about TUG1 (**D**), miR-204-5p (**E**) and COX2 mRNA (**F**) expressions and NIHSS scores respectively. Data are presented as the mean ± SD (*n* = 46 in each group). *p < 0.05, **p < 0.01, ***p < 0.001.

Table 1 shows the characteristics of the subjects. Compared with the control group, TUG1 and COX2 mRNA increased in IS patients, accompanied by a decrease in miR-204-5p (Fig. 1A–C). TUG1 and COX2 mRNA were positively correlated with NIHSS scores (Fig. 1D, F), while miR-204-5p was negatively correlated with NIHSS scores (Fig. 1E). The spatiotemporal expressions of TUG1, miR-204-5p, and COX2 mRNA were also evaluated in brain tissues of MCAO/R rats at different reperfusion time (Fig. S1D–F). These results indicated that the TUG1/miR-204-5p/COX2 axis could be involved in the CIRI occurrence and development.

**Table 1:** Basic characteristics of the subjects.

	Control (*n* = 46)	Ischemic stroke (*n* = 46)	*p*
Age (year)	64.24 ± 10.23	68.50 ± 11.25	0.0634
Male, *n* (%)	28 (60.9)	24 (52.2)	0.4002
Hypertension, *n* (%)	13 (28.2)	28 (60.9)	<0.001
Diabetes, *n* (%)	17 (36.9)	9 (20.0)	0.0640
TC (mmol/L)	4.34 ± 1.09	4.53 ± 1.26	0.4621
TG (mmol/L)	1.31 ± 0.62	1.53 ± 0.83	0.1578
LDL-C (mmol/L)	2.26 ± 0.65	2.30 ± 0.72	0.7783
HDL-C (mmol/L)	1.46 ± 0.36	1.25 ± 0.30	0.0046
NIHSS score			
1–4		18 (39.13%)	
5–15		17 (36.96%)	
16–25		11 (23.91%)	

*TC* total cholesterol, *TG* triglyceride, *LDL-C* low density lipoprotein cholesterol, *HDL-C* high density lipoprotein cholesterol, *NIHSS* the National Institutes of Health Stroke Scale.

### TUG1 knockdown ameliorated brain injury, restrained inflammatory response and apoptosis in MCAO/R rats

**Fig. 2:**
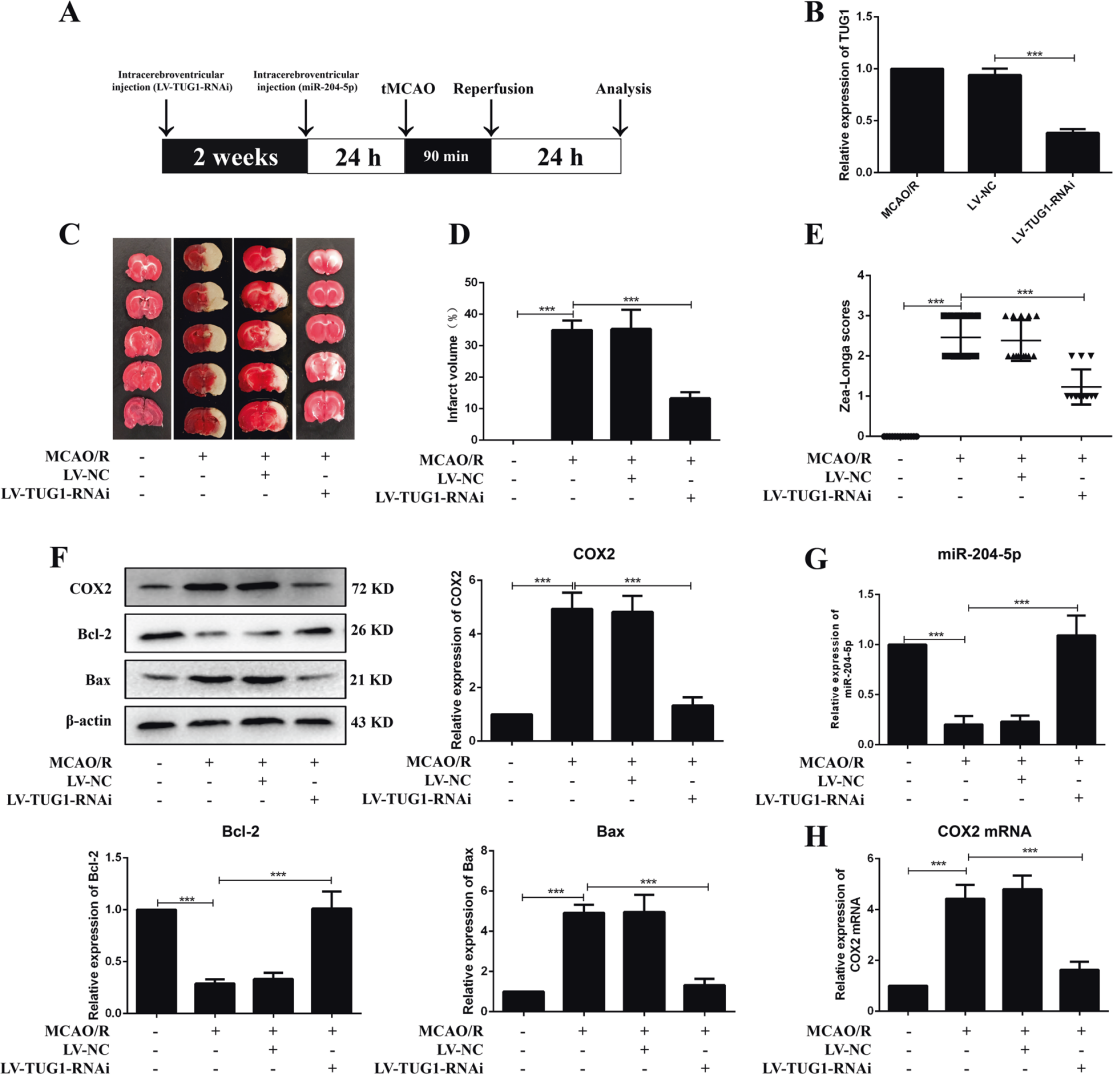
Knockdown of TUG1 ameliorated brain injury and restrained apoptosis in MCAO/R rats. **A** The diagrammatic drawing of in vivo experiment. **B** The infection efficiency of LV-TUG1-RNAi in cortex of MCAO/R rats (*n* = 6). **C** Infarct region was visualized by TTC staining. **D** Quantitative analysis of brain infarct volume after MCAO/R in rats (*n* = 9). **E** Zea-Longa scores (*n* = 13). **F** Relative protein levels of COX2, Bcl-2 and Bax in rats (*n* = 6). Relative expressions of miR-204-5p (**G**) and COX2 mRNA (**H**) in rats were detected by qRT-PCR (*n* = 6). Data are presented as the mean ± SD. ***p < 0.001.

Rats were intracerebroventricular injected with LV-TUG1-RNAi two weeks before MCAO/R operation (Fig. 2A, B). Infarct volume, Zea-Longa scores and brain injury were significantly reduced in LV-TUG1-RNAi injected rats (Figs. 2C–E and S2). The inflammatory response was inhibited after treatment with LV-TUG1-RNAi, with a decrease in COX2, IL-1β, TNF-α, and PGE2, and an increase in IL-10 (Figs. 2F, H, S3 and S4A–D). Apoptosis was restricted in LV-TUG1-RNAi group, with increased Bcl-2 and decreased Bax expression (Fig. 2F). These results suggested that TUG1 could participate in the CIRI pathogenesis by regulating the inflammatory response and apoptosis.

### miR-204-5p protected MCAO/R induced brain injury, inhibited inflammatory response and apoptosis in rats

**Fig. 3:**
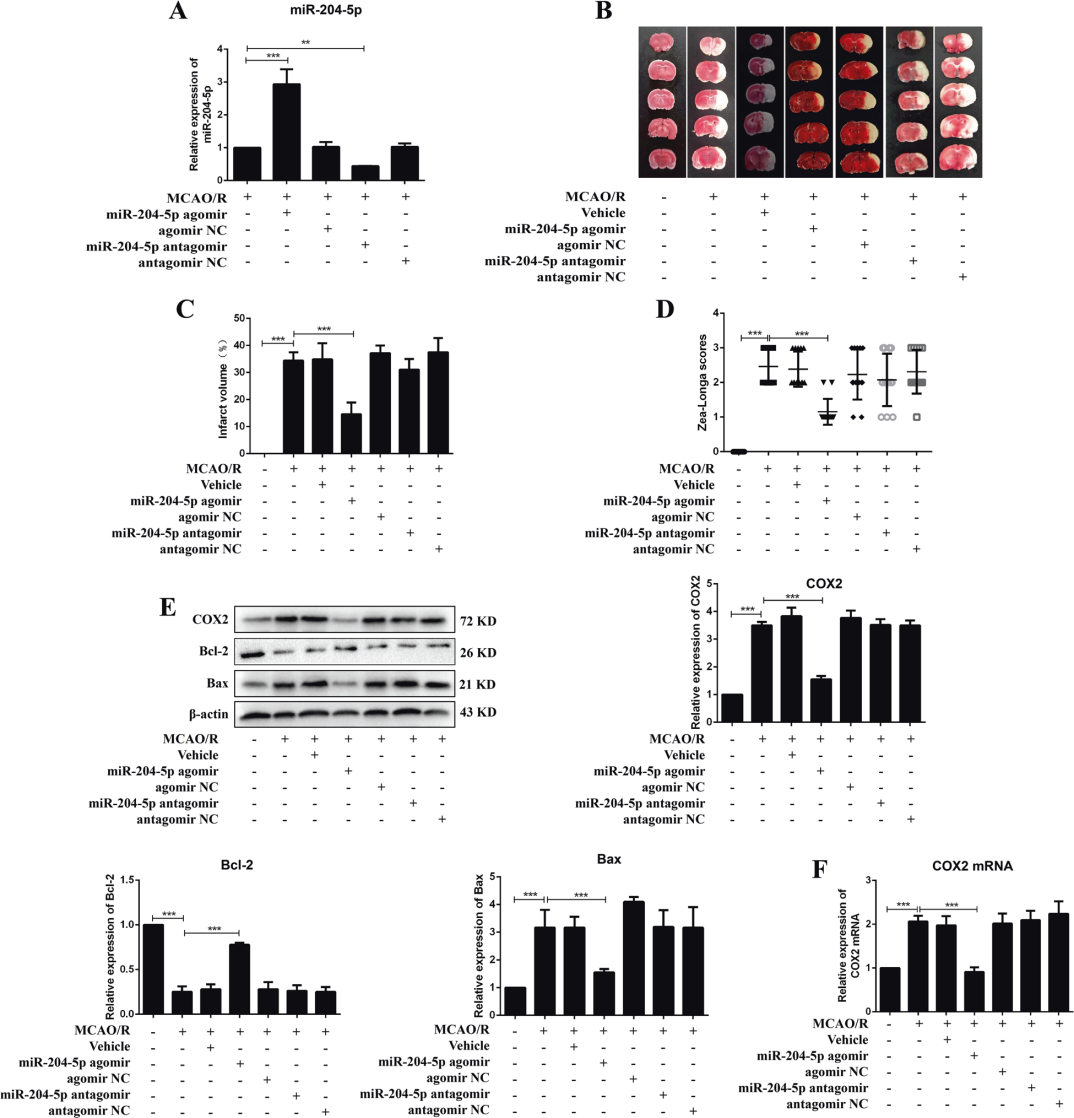
miR-204-5p improved brain injury and inhibited apoptosis in MCAO/R rats. **A** Relative expression of miR-204-5p in cortex (*n* = 6). **B** Infarct region was visualized by TTC staining. **C** Quantitative analysis of brain infarct volume after MCAO/R in rats (*n* = 9). **D** Zea-Longa scores (*n* = 13). **E** Relative protein levels of COX2, Bcl-2 and Bax in rats (*n* = 6). **F** Relative expression of COX2 mRNA in rats detected by qRT-PCR (*n* = 6). Data are presented as the mean ± SD. **p < 0.01, ***p < 0.001.

Rats were intracerebroventricular injected with miR-204-5p agomir, antagomir or negative controls 24 h before MCAO/R operation (Fig. 2A). Infarct volume and Zea-Longa scores were significantly reduced in rats treated with miR-204-5p agomir compared with the MCAO/R group (Fig. 3A–D). miR-204-5p agomir reduced COX2, IL-1β, TNF-α and PGE2 expressions, increased IL-10 expression and improved brain injury in MCAO/R rats (Figs. 3E, F, S2, S3 and S4A–D). Apoptosis was inhibited with an increase in Bcl-2 expression and a decrease in Bax expression (Fig. 3E). These results suggested that miR-204-5p protected brain injury induced by MCAO/R through inflammation and apoptosis.

### TUG1 knockdown protected neurons from OGD/R injury

**Fig. 4:**
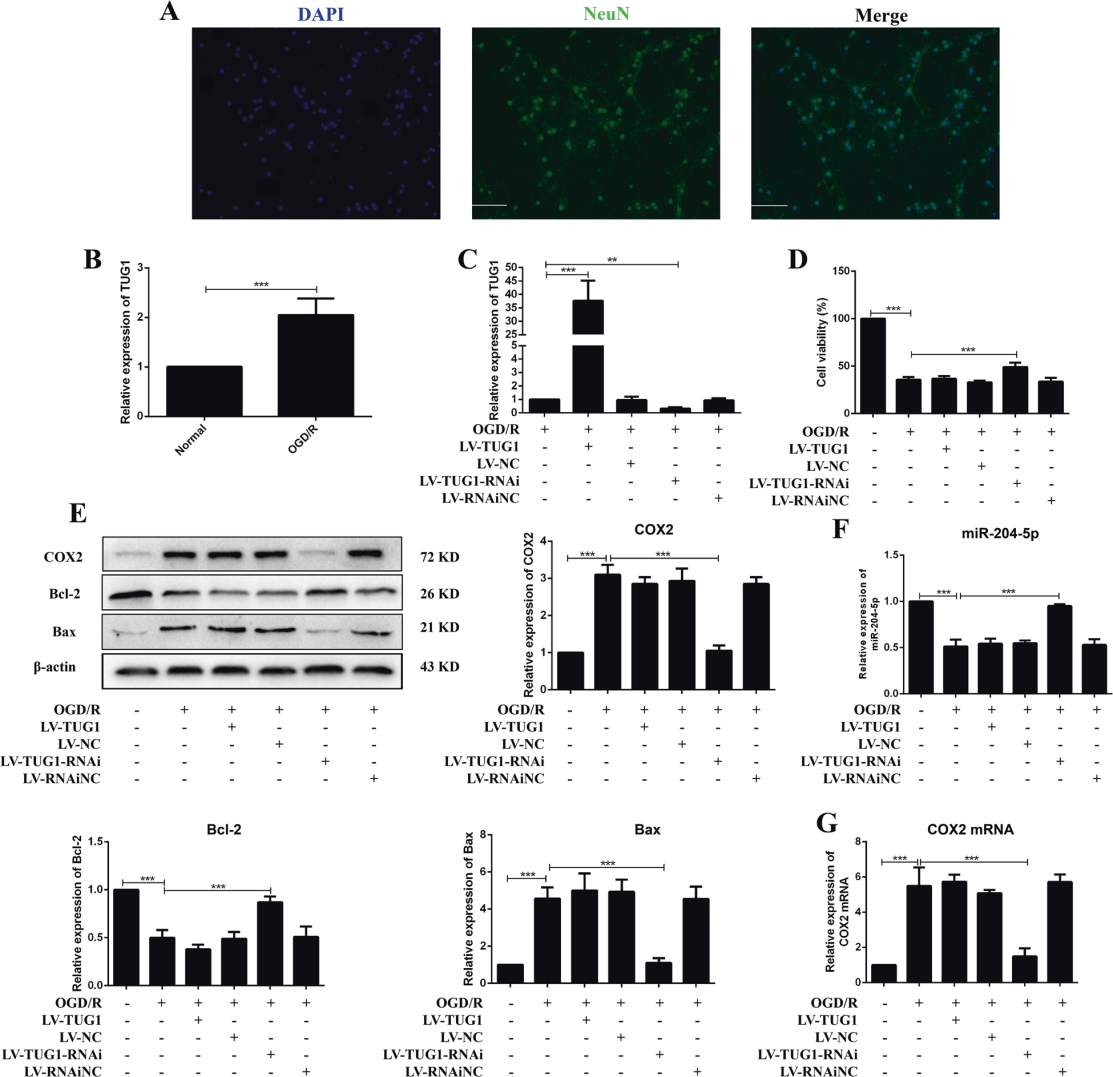
Knockdown of TUG1 protected neurons from OGD/R injury. **A** The identification of neurons was conducted by immunofluorescence. DAPI (blue), NeuN (green), 200×, scale bars = 100 μm. **B** Relative expression of TUG1 (*n* = 5). **C** The transfection efficiency of TUG1 (*n* = 5). **D** MTT assay (*n* = 6). **E** Relative protein levels of COX2, Bcl-2 and Bax in neurons (*n* = 5). Relative expression of miR-204-5p (**F**) and COX2 mRNA (**G**) in neurons (*n* = 5). Data are presented as the mean ± SD. **p < 0.01, ***p < 0.001.

Primary cortical neurons were isolated from the cortex of SD rats and were exposed to OGD/R to simulate ischemia and reperfusion injury in vitro (Fig. 4A). Compared with the normal group, TUG1 was significantly upregulated in the OGD/R group (Fig. 4B). TUG1 overexpression and suppression in neurons were achieved by transfection with LV-TUG1 or LV-TUG1-RNAi, respectively (Fig. 4C). The impairment of cell viability in neurons treated with OGD/R was alleviated after TUG1 knockdown (Fig. 4D). Apoptosis and inflammation were inhibited in OGD/R neurons transfected with LV-TUG1-RNAi (Figs. 4E, G and S6). These results demonstrated that TUG1 knockdown protected neurons from OGD/R injury.

### miR-204-5p promoted neuron survival in OGD/R

**Fig. 5:**
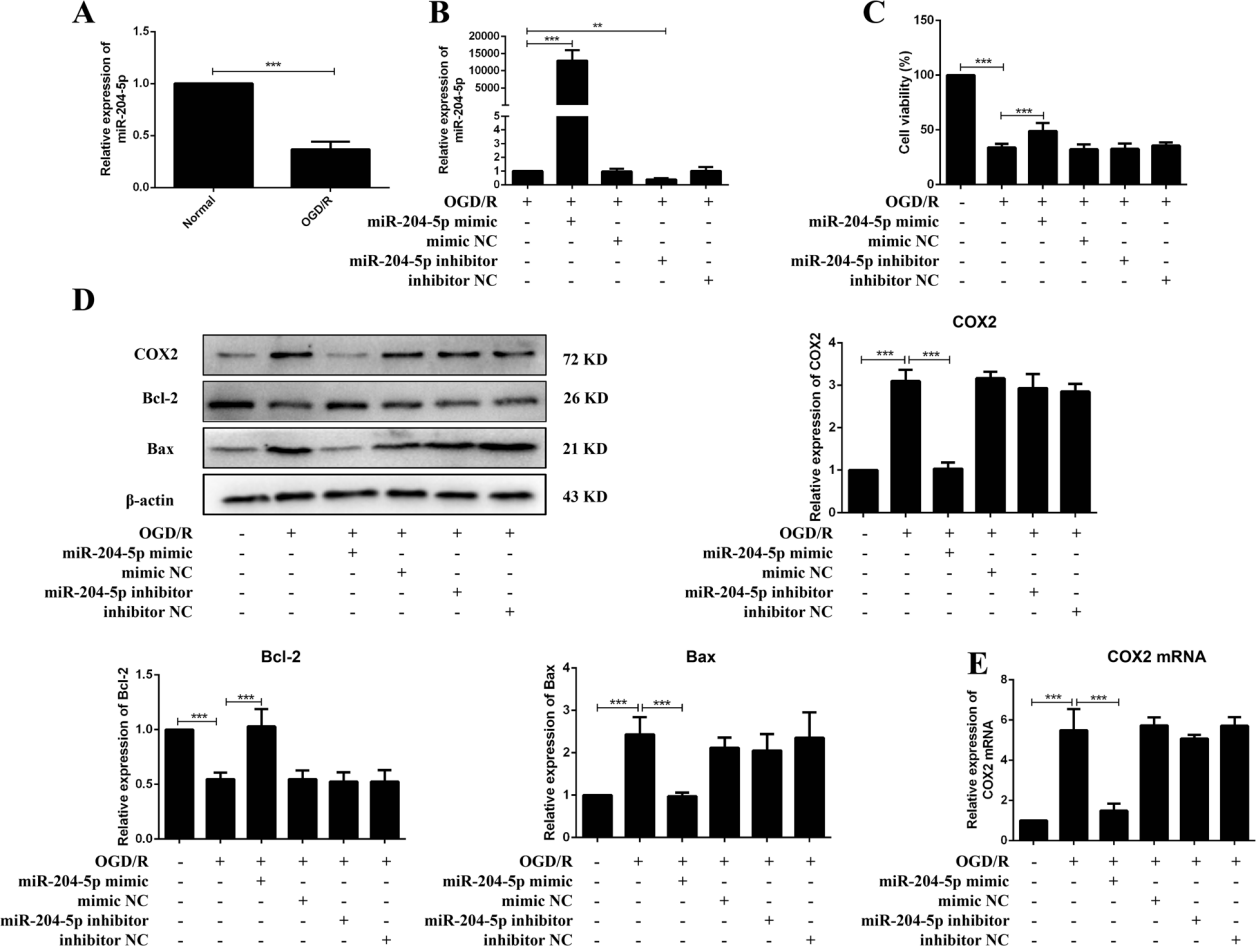
miR-204-5p promoted neuron survival in OGD/R. **A** Relative expression of miR-204-5p (*n* = 5). **B** The transfection efficiency of miR-204-5p (*n* = 5). **C** MTT assay (*n* = 6). **D** Relative protein levels of COX2, Bcl-2 and Bax in neurons (*n* = 5). **E** Relative expression of COX2 mRNA in neurons (*n* = 5). Data are presented as the mean ± SD. **p < 0.01, ***p < 0.001.

miR-204-5p was elevated with TUG1 knockdown both in vivo and in vitro (Figs. 2G and 4F). miR-204-5p was negatively regulated in OGD/R neurons (Fig. 5A). The expression of miR-204-5p on OGD/R injury after neurons were transfected with miR-204-5p mimics or inhibitors (Fig. 5B). The impairment of cell viability in OGD/R neurons improved after transfection with miR-204-5p mimics (Fig. 5C). Apoptosis and inflammation were inhibited in OGD/R neurons pretreated with miR-204-5p mimics (Figs. 5D and S6). Decreased expressions of COX2 mRNA and protein were detected in the miR-204-5p mimic group than the OGD/R group (Figs. 5D and 5E). These results demonstrated that miR-204-5p promoted neuron survival in OGD/R.

### TUG1 knockdown inhibited inflammation and apoptosis by up-regulating miR-204-5p

Cell viability was improved in the LV-TUG1 + miR-204-5p mimic group compared with the LV-TUG1 group, along with increased expression of miR-204-5p (Fig. S5A and C). Apoptosis and inflammation were alleviated in the LV-TUG1 + miR-204-5p mimic group (Figs. S5B and S6). Cell viability was damaged in the LV-TUG1-RNAi + miR-204-5p inhibitor group than the LV-TUG1-RNAi group, along with a decreased expression of miR-204-5p (Fig. S5D and F). Apoptosis and inflammation were aggravated in the LV-TUG1-RNAi + miR-204-5p inhibitor group (Figs. S5E and S6). Decreased COX2 and Bax protein levels and increased Bcl-2 protein levels were reversed when LV-TUG1-RNAi was co-infected with miR-204-5p antagomir in vivo, together with a reduced expression of miR-204-5p (Figs. S3 and S7A–C). The expressions of IL-1β, TNF-α, PGE2, and IL-10 were reversed in MCAO/R rats co-infected with LV-TUG1-RNAi + miR-204-5p antagomir (Figs. S4A–D). These results implied that TUG1 knockdown inhibited inflammation and apoptosis by upregulating miR-204-5p.

### miR-204-5p targeted COX2 mRNA 3′-UTR and downregulated its expression

**Fig. 6:**
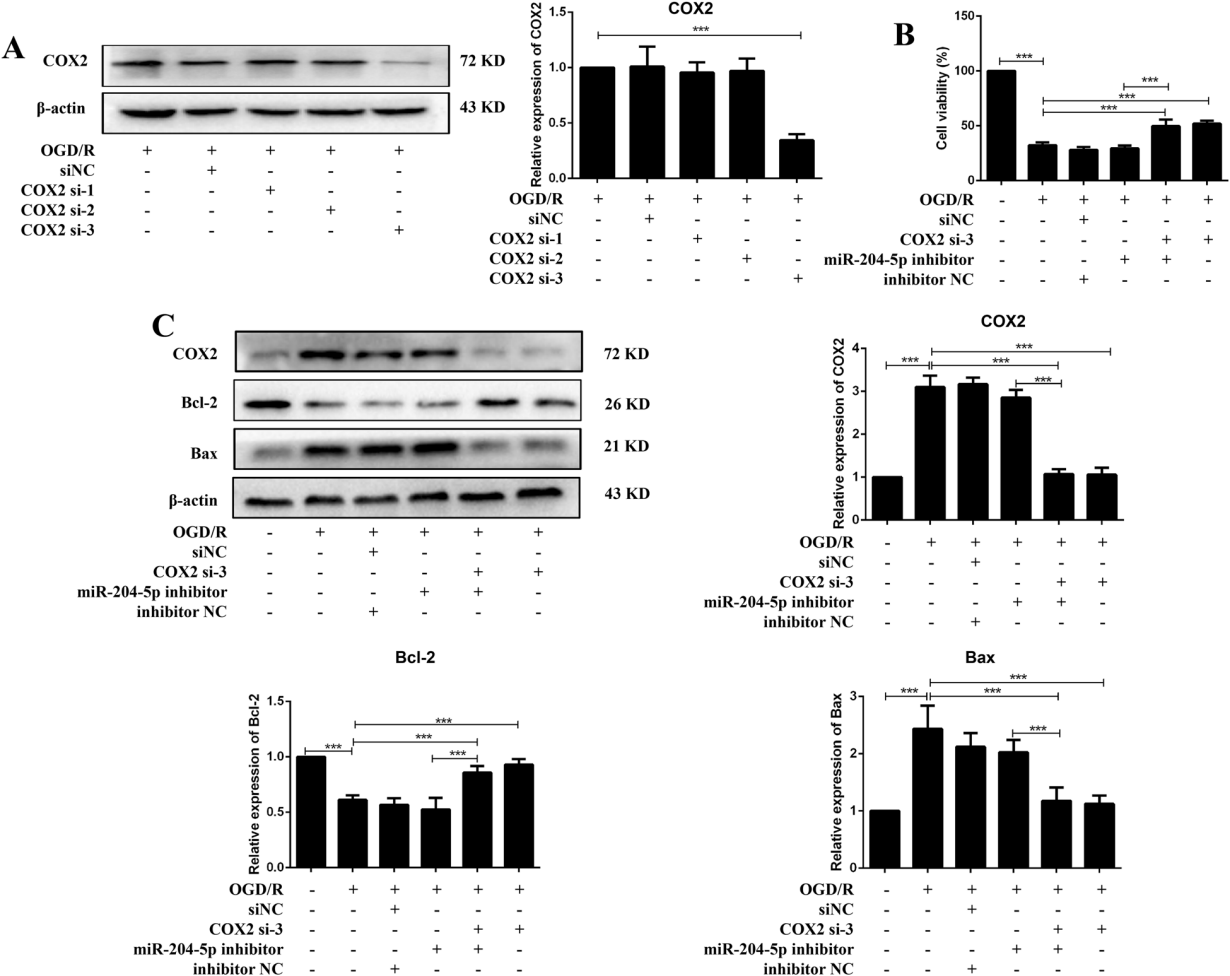
COX2 was modulated by both TUG1 and miR-204-5p. **A** Relative expression of COX2 after transfected with COX2 siRNAs in neurons (*n* = 5). **B** MTT assay was performed to assess cell viability after co-transfected with miR-204-5p inhibitor and COX2 si-3 (*n* = 6). **C** Relative protein levels of COX2, Bcl-2 and Bax in neurons after co-transfected with miR-204-5p inhibitor and COX2 si-3 (*n* = 5). Data are presented as the mean ± SD. ***p < 0.001.

**Fig. 7:**
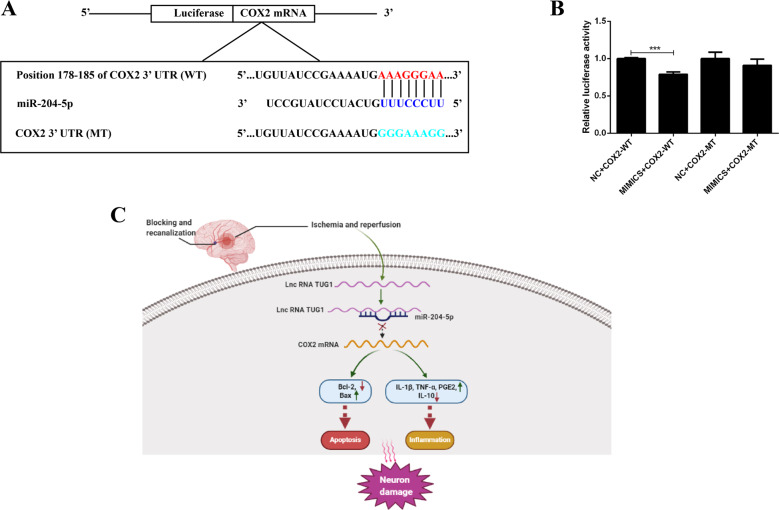
COX2 was a target of miR-204-5p. **A** Presentation of the putative binding site of miR-204-5p and COX2, and the designed mutant sequence. **B** The relative luciferase activity of HEK 293 cells co-transfected COX2-WT or COX2 MT with miR-204-5p mimic or NC (*n* = 5). **C** The illustration of how TUG1/miR-204-5p/COX2 axis was involved in ischemia and reperfusion induced neuron injury. Data are presented as the mean ± SD. ***p < 0.001.

COX2 expression was negatively correlated with miR-204-5p in vivo and vitro (Figs. 3E and 5D). Three small interfering RNAs of COX2 were transfected into neurons, respectively (Fig. 6A). The decreased cell viability induced by the miR-204-5p inhibitor was negated by co-transfection with COX2 si-3 in OGD/R neurons (Fig. 6B), and increased apoptosis and inflammation were moderated by co-transfection with COX2 si-3 (Figs. 6C and S6). Figure 7A shows the predicted latent binding site between miR-204-5p and COX2 mRNA 3’-UTR. Cells transfected with miR-204-5p mimics and COX2 WT showed significantly reduced relative luciferase activity than other groups (Fig. 7B). These results supported that COX2 was a downstream target of the TUG1/miR-204-5p pathway in CIRI (Fig. 7C).

## Discussion

The injury of neurons contributes to the secondary brain ischemic injury and represents the leading cause of cerebral injury aggravation [27]. Therefore, we focused on the mechanism of neuron injury involved in CIRI. We found that miR-204-5p improved ischemia and reperfusion-induced neuron injury by downregulating COX2 expression.

Our previous study has shown that COX2 is upregulated in CIRI, and miR-211-5p could alleviate CIRI in rats by downregulating the expression of COX2 [27]. miR-204-5p and miR-211-5p have very similar nucleotide sequences with only one different nucleotide in the entire sequence, a plausible explanation for sharing common targets [28]. In the previous study, we detected that miR-204-5p was decreased in the MCAO/R model, while the exact mechanism of neuron injury has not been elaborated. This study confirmed that miR-204-5p was downregulated in the blood of IS patients and the brains of MCAO/R rats.

The modulating network in organisms is complex and other genes can regulate miR-204-5p. TUG1 was first identified to play an essential role in the formation of photoreceptors [29, 30]. TUG1 sponges microRNA-9 to promote neuronal apoptosis by upregulating Bcl2l11 under ischemia, indicating the therapeutic potential of TUG1 in IS [25]. TUG1 knockdown significantly reduced the infarct volume and Zeal-Long scores, promoted cell viability, and decreased apoptosis in OGD/R neurons. miR-204-5p antagomir or inhibitors could abolish the improvement of CIRI after TUG1 knockdown. Our study revealed that TUG1 acted as a regulator of miR-204-5p in CIRI.

When the brain suffers from ischemia and reperfusion, COX and other enzymes that promote ROS production destroyed the dynamic balance of ROS and caused neuron injury [31]. The suppression of TUG1 or overexpression of miR-204-5p enhanced the SOD content and weakened the expression of COX2, effectively reducing damage from oxidative stress (Fig. S4E). MDA is cytotoxic, causing the cross-linking and polymerization of macromolecules such as proteins and nucleic acids. Our results showed that MDA increased significantly in MCAO/R rats (Fig. S4F). MDA is the final product of the lipid oxidation reaction and is regulated by many different enzymes that promote or inhibit ROS production. The toxic effect of COX2 appears to be mediated by PGE2 rather than ROS, although COX2 can generate both [32]. Therefore, the simple change in COX2 and SOD would not significantly affect the MDA content.

Studies have shown that IL-1β, IL-10, and TNF-α in brain tissues are produced mainly by inflammatory cells [33]. We did not detect IL-1β, IL-10 and TNF-α in primary cortical neurons. PGE2 is the primary PGE product of COX2 in the brain, specifically derived from the intermediate product PGH2 catalyzed by COX2. Under pathological conditions, PGH2 is further catalyzed into PGE2 by microsomal PGE synthase 1 (mPGES-1) [34]. PGE2 is an important mediator to mediate harmful effects in neurological diseases [35, 36]. Due to the rapid induction of COX2 and mPGES-1, the level of PGE2 increases correspondingly after cerebral ischemia. At 24 h after MCAO, the PGE2 level of the ipsilateral cortex in mice was three times higher than that of the contralateral cortex [37]. Our results showed that TUG1 knockdown or miR-204-5p overexpression significantly reduced PGE2 content.

In conclusion, our study suggested that decreased miR-204-5p promoted the inflammatory response and apoptosis and aggravated the neuronal damage induced by CIRI. The TUG1/miR-204-5p/COX2 axis was involved in CIRI. This finding may provide a novel strategy for treating CIRI.

## Materials and methods

### Sample collection

The sample size was calculated based on our pre-experiments. A 1.4 (SD 2) relative difference was assumed in the relative expression of miR-204-5p between healthy volunteers and IS patients. At least 44 subjects in each group were needed (90% power, 5% significance) with a two-tailed test. Forty-six patients who suffered an initial IS were recruited from the Department of Neurology of Dianjiang People’s Hospital of Chongqing (DPHC) from December 2019 to May 2020. Patients with a history of hemorrhagic stroke, peripheral artery occlusive disease, transient ischemic attack, impaired consciousness or epilepsy, severe lung, liver or kidney dysfunction, severe malnutrition, thyroid disease, primary or metastatic tumors, and those who were pregnant or breastfeeding were excluded. Blood samples were collected within two hours after patient admission and stored at −80 °C until RNA extraction. Forty-six healthy volunteers from DPHC were enrolled. The experiment was approved by the DPHC Ethics Committee. All subjects or their legal representatives signed a written informed consent.

### Animals and the middle cerebral artery occlusion/reperfusion (MCAO/R) model

Adult male Sprague Dawley (SD) rats weighing 250–280 g (8–10 weeks old) were obtained and fed at the Laboratory Animal Center, Chongqing Medical University, China (license number: SYXK YU 2012-0001). The rats were kept at 23 °C and 70% humidity in a 12 h light/dark cycle with free access to food and water, and randomized by lottery without blinding. Considering statistical requirements and the minimization of experimental animals, at least fifteen rats were included in each group. All animal experiments were performed according to the center guidelines and in accordance with the Ethics Committee of Chongqing Medical University.

Rats were first anesthetized with an intraperitoneal injection of 3% pentobarbital sodium (1 ml/kg). Then the common carotid artery (CCA), internal carotid artery (ICA), and external carotid artery (ECA) were carefully isolated. The CCA was blocked by a bulldog clamp. The ICA was intercepted by a suture line. A silicone rubber coated nylon monofilament was inserted into the ECA until it reached the origin of the middle cerebral artery. After 90 min of occlusion, the monofilament was removed for reperfusion. The rats were deeply anesthetized and brains were immediately removed for further analysis after reperfusion. The sham group underwent the same procedure without occlusion. Body temperature was maintained at 37 ± 0.5 °C during operation.

### Lateral intracerebroventricular injection

Rats were anesthetized and the right lateral cerebral ventricle was exposed to a stereotaxic apparatus. The coordinates were 0.8 mm anteroposterior, 1.5 mm lateral, and 3.5 mm dorsoventral to the bregma [38]. LV-TUG1-RNAi (1 × 10^8^ TU/mL, GeneChem, China) or negative control were slowly injected into the lateral ventricle in 10 min (2.5 µL). The rats were then fed for 14 days and subjected to MCAO/R. miR-204-5p agomir, antagomir, and their negative controls were synthesized by GenePharma (Shanghai, China) and diluted in artificial cerebrospinal fluid (Dingguo, China). One day before the MCAO/R operation, 5 µL (100 µM) miR-204-5p agomir, antagomir, or heir negative controls were injected into the right lateral ventricle, respectively. The rats were sacrificed the next day to obtain brain tissue.

### Neurological deficit assessment

Neurological deficit assessment was performed after reperfusion for 24 h using the Zea-Longa method: 0 points, no behavior disorder; 1 point, flexion of the left forelimb; 2 points, body turns to the left in a circle; 3 points, dump to the left; and 4 points, inability to walk autonomously with disturbance of consciousness.

### Infarct volume measurement

The freezing brains were cut into slices and stained with 2% 2,3,5-triphenyl tetrazolium chloride (TTC, Sigma, St. Louis, USA) dissolved in phosphate-buffered solution (PBS) for 20 min at 37^o^C. The stained slices were fixed with 4% paraformaldehyde dissolved in PBS (0.1 M, pH 7.2) and photographed with a camera. Infarct volume (%) was measured using Image J software (Bethesda, USA) using the formula: volume of the infarct area/volume of total brain × 100%.

### Hematoxylin-eosin staining

The rats were anesthetized and transcardially perfused with 100 mL PBS and fixed with 50 mL of 4% paraformaldehyde. The whole brain was removed and stored in 4% paraformaldehyde, cut into 5 μm thick coronal sections, and then stained with hematoxylin and eosin solutions. The images were captured by a microscope (Olympus, Tokyo, Japan).

### Superoxide dismutase (SOD) and malondialdehyde (MDA) assay

Brain tissues were thoroughly ground in cold saline and the suspension was centrifuged at 3500 rpm for 10 min. The SOD activity and MDA content were detected by commercial kits (Nanjing Jiancheng, China).

### Cell cultures

Primary cortical cultures were prepared from E17 embryos from SD rats [39]. The cerebral cortex of the embryos was isolated and chopped immediately and then incubated with 0.25% trypsin-EDTA (Gibco, USA) at 37 °C for 15 min. Digestion was stopped with Dulbecco’s modified Eagle Medium/Ham’s F-12 containing 10% fetal bovine serum (Biological Industries, Israel). The suspension was filtered with a 200 mesh screen and centrifuged for 5 min at 1000 r/min. Cells were suspended in neurobasal medium (Gibco, USA) supplemented with 2% B27 (Gibco, USA), 0.5 mM l-glutamine (Gibco, USA) and 1% penicillin–streptomycin (10,000 U/ml, Gibco, USA), and seeded at a density of 2 × 10^5^ cells/cm^2^ in plates precoated with poly-L-lysine (0.1 mg/ml, Sigma–Aldrich, USA). Cells were cultured at 37 °C in humidified air (5% CO_2_/95%) and the medium was changed half every three days.

### Oxygen and glucose deprivation and re-oxygenation

Cell medium was replaced with glucose-free DMEM (Gibco). Cells were incubated in an anaerobic incubator for 1 h, and then placed in a complete neurobasal medium in normoxia for another 24 h.

### Cell transfection

LV-TUG1-RNAi (1 × 10^8^ TU/ml), LV-TUG1 (2 × 10^7^ TU/ml) and negative controls were synthesized by GeneChem. Cells were transfected with HiTransG A (GeneChem) three days before OGD. miR-204-5p mimics, inhibitors, COX2 siRNA1, siRNA2, siRNA3, and their negative controls were synthesized by GenePharma. Cells were transfected with TransIntro^TM^ EL transfection reagent (TransGen, China) one day before OGD at a concentration of 10 nM.

### Cell viability assay

The survival of neurons with OGD/R treatment was evaluated using the 3-(4,5-dimethylthiazol-2-yl)-2,5-diphenyltetrazolium bromide (MTT) assay. MTT solution (5 mg/mL, Sigma-Aldrich) was added to each well and incubated at 37^o^C for 4 h. The supernatant was discarded and the formazan was dissolved in 150 μL dimethyl sulfoxide. The optical density of each well was determined using a microplate reader (Thermo Scientific, USA) at 490 nm.

### Quantitative real-time PCR

Total RNA was extracted using a trizol reagent (Vazyme, China). RNAs were reverse transcribed to cDNA and miRNA using an all-in-one cDNA synthesis supermix (Bimake, USA) and a miRNA first-strand cDNA synthesis reagent kit (Sangon, China). Expressions of mRNA and miRNA were measured using the 2 × SYBR Green qPCR Master Mix (Bimake). The primer sequences are shown in Table S1.

### Western blot

Brain tissues and cells were ground in ice-cold RIPA buffer supplemented with a 1% cocktail (Bimake) to extract total protein. The supernatant was quantified using the Bradford Protein Kit (Servicebio, China). Proteins were separated on 12.5% SDS-PAGE and transferred to polyvinylidene difluoride (PVDF) membranes (Millipore, USA). The membranes were incubated with primary antibodies at 4 °C overnight, including COX2 (#ab15191, 1:1000, Abcam, UK), Bcl-2 (#AF6139, 1:1000, Affinity, USA), Bax (#60267-1-Ig, 1:10000, Proteintech, USA), and β-actin (#A5538, 1:2000, Bimake). HRP-conjugated secondary antibodies (#SA00001-1 and #SA00001-2, 1:2000, Proteintech, USA) were incubated for another 1 h at room temperature. Immunoblots were visualized using an enhanced chemiluminescence detection system (Bio-rad, USA). Relative integrated density values were calculated using Image Lab software.

### Immunofluorescence staining

Brains were dehydrated in graded alcohol and embedded in paraffin. The brains were cut into 5 μm thick coronal sections. Slices were blocked with 5% goat serum for 2 h and incubated with rabbit polyclonal anti-COX2 antibody (#ab15191, 1:200, Abcam) and mouse monoclonal anti-NeuN antibody (#GTX30773, 1:200, GeneTex, USA) overnight at 4 °C. The sections were incubated with Alexa 488-labeled goat anti-rabbit IgG (#A0423, 1:200, Beyotime, China) and Cy3-labeled goat anti-mouse IgG (#A0521, 1:200, Beyotime) for 1 h at 37 °C. The nuclei were stained with 4, 6-diamidino-2-phenylindole (DAPI, Beyotime) for 5 min at room temperature. The cells were imaged by an Olympus fluorescence microscope.

Neurons were fixed with 4% paraformaldehyde, permeabilized with 0.3% Triton X-100 in PBS and blocked with 5% goat serum for 2 h. The remaining steps were consistent with the method mentioned above.

### Apoptosis analysis

Cell apoptosis was evaluated using the Fluorescein (FITC) tunel cell apoptosis detection kit (Servicebio, China). Cells were gently washed with PBS and fixed with 4% paraformaldehyde. The cells were then permeabilized with 0.1% Triton X-100 for 20 min. A 50 μL equilibration buffer was added and incubated at room temperature for 30 min. The balanced calibration buffer was removed and cells were incubated in 56 μL TdT incubation buffer at 37 ^o^C for 2 h. The nuclei were then stained with DAPI for 8 min at room temperature. The cells were imaged by an Olympus fluorescence microscope.

### Enzyme-linked immunosorbent (ELISA) assay

Cell culture supernatant was centrifuged at 3500 rpm for 10 min. The content of IL-1β, IL-10, TNF-α, and PGE2 was detected using the ELISA kit (Meibiao, China).

### Dual-luciferase reporter gene assays

The supposed binding site of miR-204-5p and COX2 3′-UTR was cloned by PCR and inserted into a pmirGLO vector to create COX2 WT. The corresponding mutants COX2 MT were constructed according to the supposed binding site. HEK 293 cells were transfected with pmirGLO vectors and miR-204-5p mimic by lipofectamine 2000 (Thermofisher, USA). After 48 h, dual luciferase detection was performed using the Dual-Luciferase Reporter Gene Assay Kit (Promega, USA). Firefly luciferase activity was normalized to Renilla luciferase activity.

### Statistical analysis

All data were expressed as mean ± standard deviation (SD). Data were from at least three independent experiments and analyzed using GraphPad Prism 5. A single comparison between two groups was analyzed by an independent *t*-test, and multiple group comparisons were analyzed with a one-way analysis of variance (ANOVA). The association of the two variables was evaluated using a Pearson correlation analysis with two-tailed test, with *p* < 0.05 considered significant.

## Supplementary information

supplementary information

Original WB images

Reproducibility Checklist

## Data Availability

The datasets are available from the corresponding author on reasonable request. Supplementary information is available at Cell Death Discovery’s website.
