# Supplementary material for: miR-204-5p is sponged by TUG1 to aggravate neuron damage induced by focal cerebral ischemia and reperfusion injury through upregulating COX2
Source: Cell Death Discov. 2022 Feb 28;8:89. doi: 10.1038/s41420-022-00885-x (PMC8885635; doi:10.1038/s41420-022-00885-x)
Supplement: Supplementary file 1 — supplementary information [file 41420_2022_885_MOESM1_ESM.docx]

**miR-204-5p is sponged by TUG1 to aggravate neuron damage induced by focal cerebral ischemia and reperfusion injury through upregulating COX2**

Pu Xiang^1,2,#^, Jian Hu^3,#^, Hong Wang^1^, Ying Luo^1^, Chao Gu^1^, Xiaodan Tan^1^, Yujun Tu^1^, Wenjia Guo^1^, Lin Chen^4^, Lin Gao^4^, Rongchun Chen^1^ and Junqing Yang^1,*^.

^1^ Department of Pharmacology, Chongqing Medical University, the Key Laboratory of Biochemistry and Molecular Pharmacology, Chongqing 400016, China

^2^ Department of Pharmacy, Dianjiang People's Hospital of Chongqing, Chongqing 408300, China

^3^ Department of Hepatobiliary Surgery, Dianjiang People's Hospital of Chongqing, 408300, China

^4^ Department of Neurology, Dianjiang People's Hospital of Chongqing, Chongqing 408300, China

^*^Corresponding author:

Prof. Junqing Yang,

Department of Pharmacology, Chongqing Medical University,

Chongqing 400010, China;

Tel: +86-23-68485161; Fax: +86-23-68485161; E-mail: cqyangjq@cqmu.edu.cn

The authors declared that they have no conflicts of interest with this work.

#: Pu Xiang and Jian Hu are the co-first authors.

Table S1 List of primer sequences in qRT-PCR analysis.

| Gene | Forward (5’-3’) | Reverse (5’-3’) |
| --- | --- | --- |
| hsa-TUG1 | TAGCAGTTCCCCAATCCTTG | CACAAATTCCATCATTCCC |
| hsa-COX2 | TGTCAAAACCGAGGTGTATGTA | AACGTTCCAAAATCCCTTGAAG |
| rno-TUG1 | GGCGTATAGAAGGTTGGCAGCAG | ACTTGGCAAGCAGGTCTGTGATG |
| rno-COX2 | AGACAGATCAGAAGCGAGGACCTG | ATACACCTCTCCACCGATGACCTG |
| miR-204-5p | CGCTTCCCTTTGTCATCCTATGCCT |  |
| U6 | CCTGCTTCGGCAGCACA | AACGCTTCACGAATTTGCGT |
| hsa-ACTB | CCTGGCACCCAGCACAAT | GGGCCGGACTCGTCATAC |
| rno-ACTB | TGTCACCAACTGGGACGATA | GGGGTGTTGAAGGTCTCAAA |


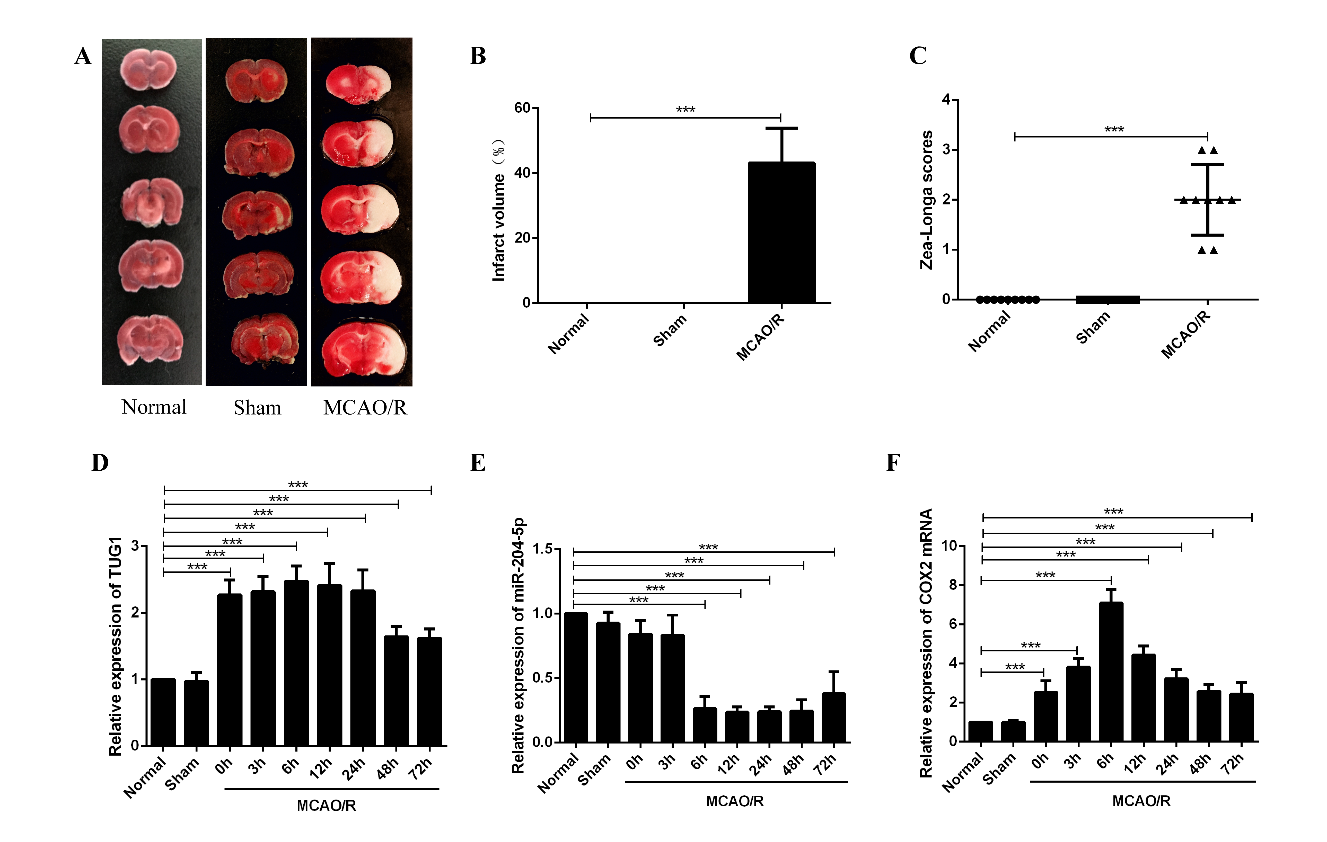


Figure S1 The spatio-temporal expressions of TUG1, miR-204-5p and COX2 mRNA in MCAO/R rats. **A** Infarct region was visualized by TTC staining. **B** Quantitative analysis of brain infarct volume after MCAO/R in rats. (n = 9) **C** Zea-Longa scores. (n = 9) The expressions of TUG1 (**D**), miR-204-5p (**E)** and COX2 mRNA (**F**) in MCAO rats detected by qRT-PCR after reperfusion for 0, 3, 6, 12, 24, 28 and 72 h respectively. (n = 6) Data are presented as the mean ± SD. ^***^p<0.001.


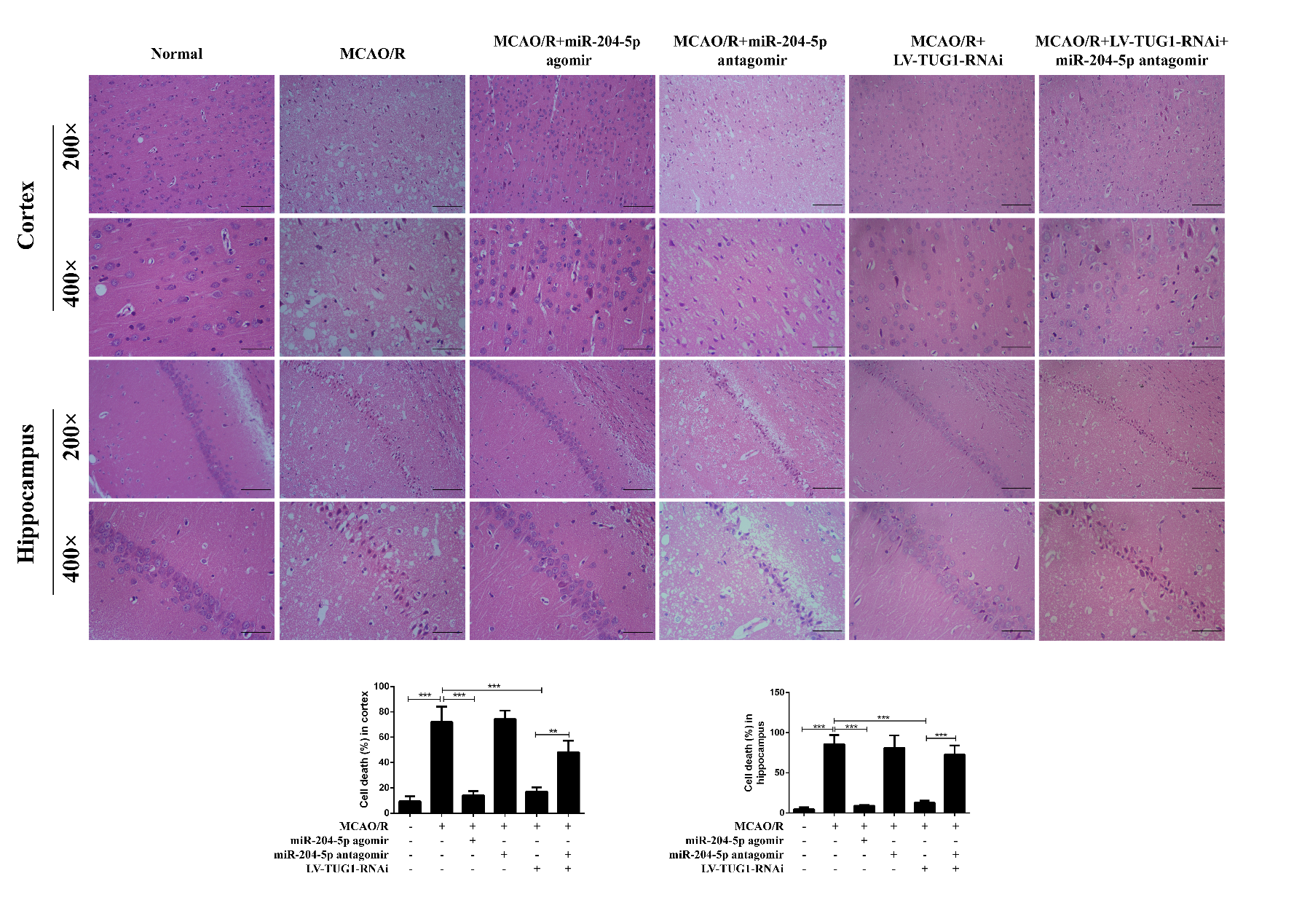


Figure S2 Representative images of hematoxylin-eosin (HE) staining in the cortex and hippocampus of rats. 200×, scale bars = 100 μm; 400×, scale bars = 50 μm. Data are presented as the mean ± SD (n = 3 in each group). ^**^p<0.01, ^***^p<0.001.


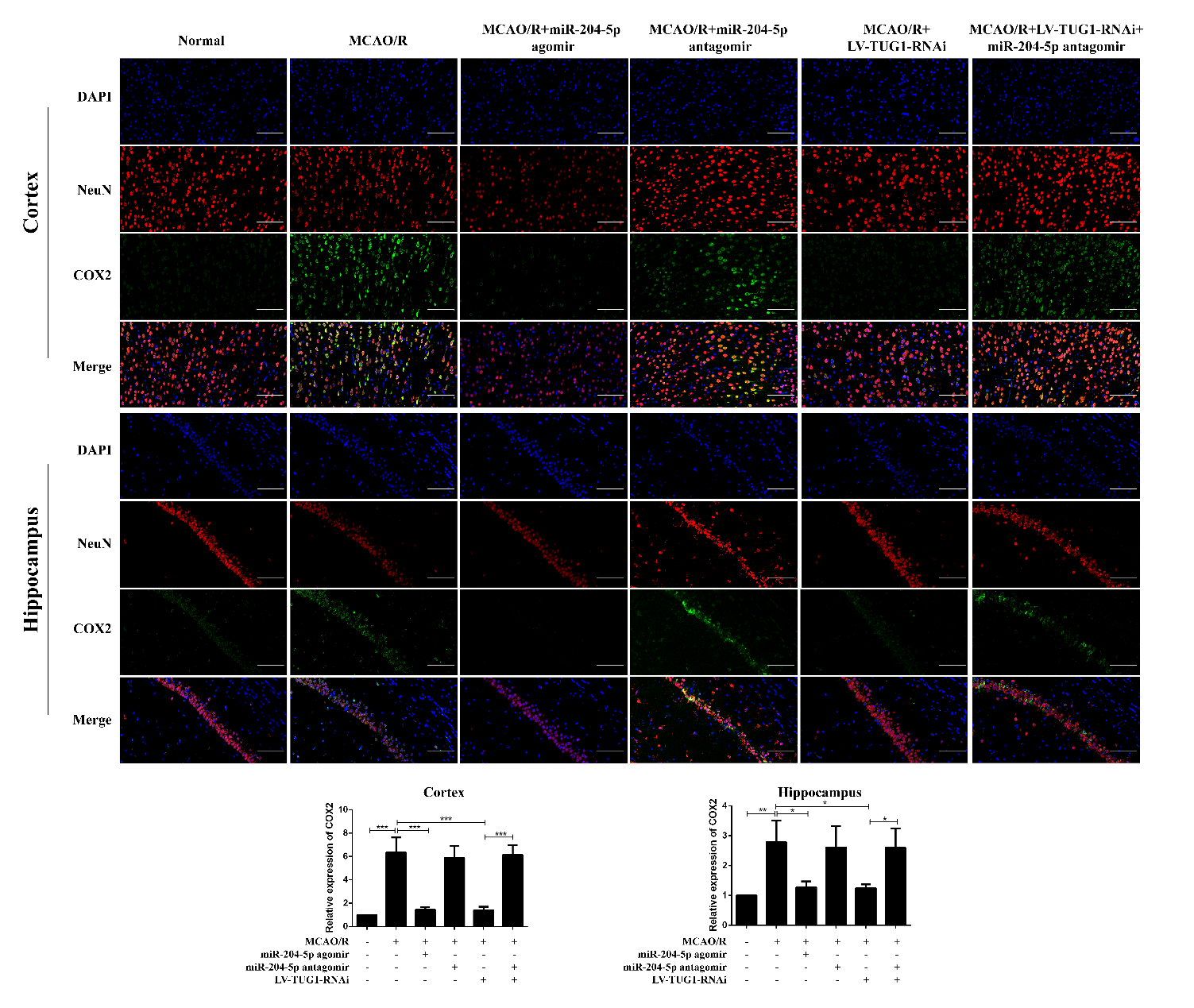


Figure S3 The expression of COX2 was co-localization with neurons both in cortex of hippocampus in rats by immunofluorescent staining. DAPI (blue), NeuN (red), COX2 (green), 400×, scale bars = 50 μm. Data are presented as the mean ± SD (n = 3 in each group). ^##^p<0.01 vs. normal group, ^###^p<0.001 vs. normal group. ^*^p<0.05, ^**^p<0.01, ^***^p<0.001.


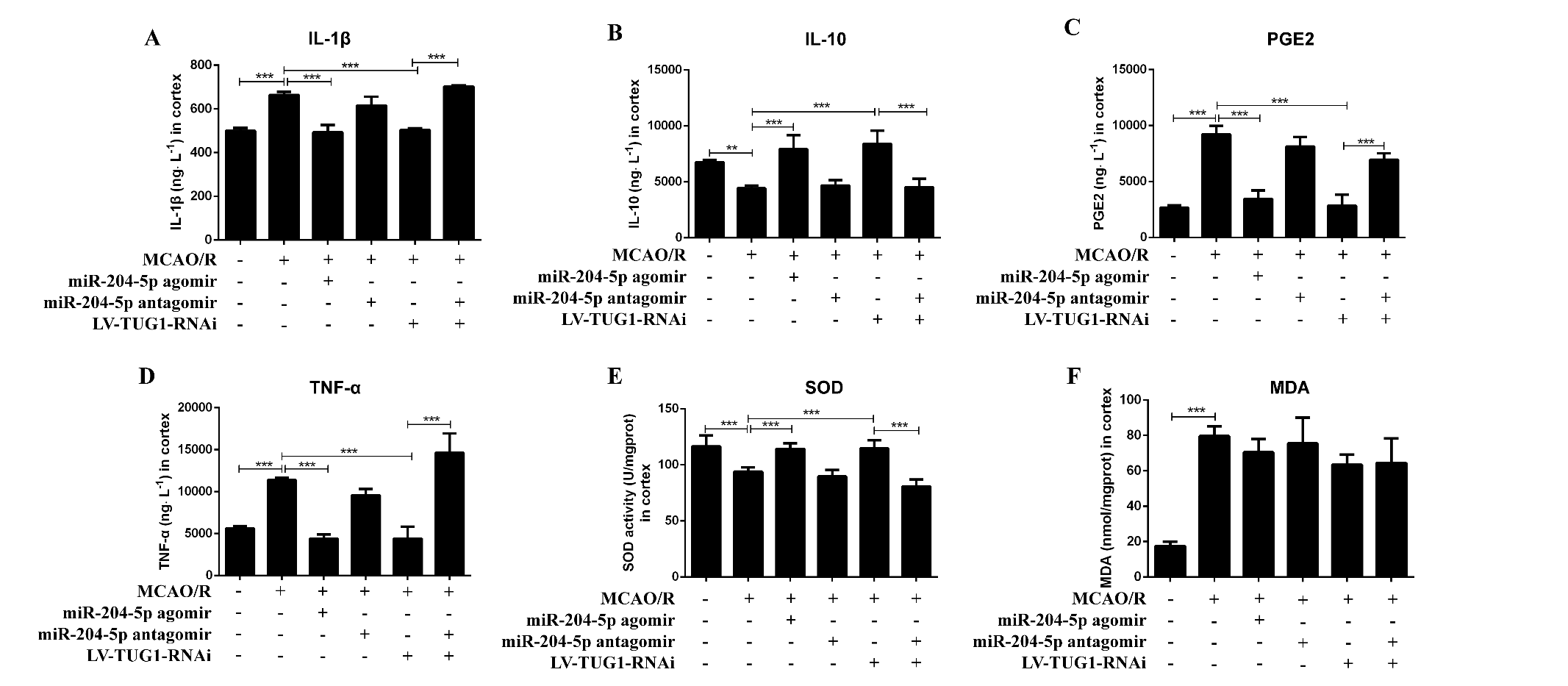


Figure S4 TUG1 and miR-204-5p regulated inflammation and oxidative stress in MCAO/R rats. The levels of IL-1β (**A**), TNF-α (**B**), PGE2 (**C**) and IL-10 (**D**) were detected by ELISA kits. SOD activity (**E**) and MDA (**F**) content were detected in the cortex of rats. Data are presented as the mean ± SD (n = 5 in each group). ^**^p<0.01, ^***^p<0.001.


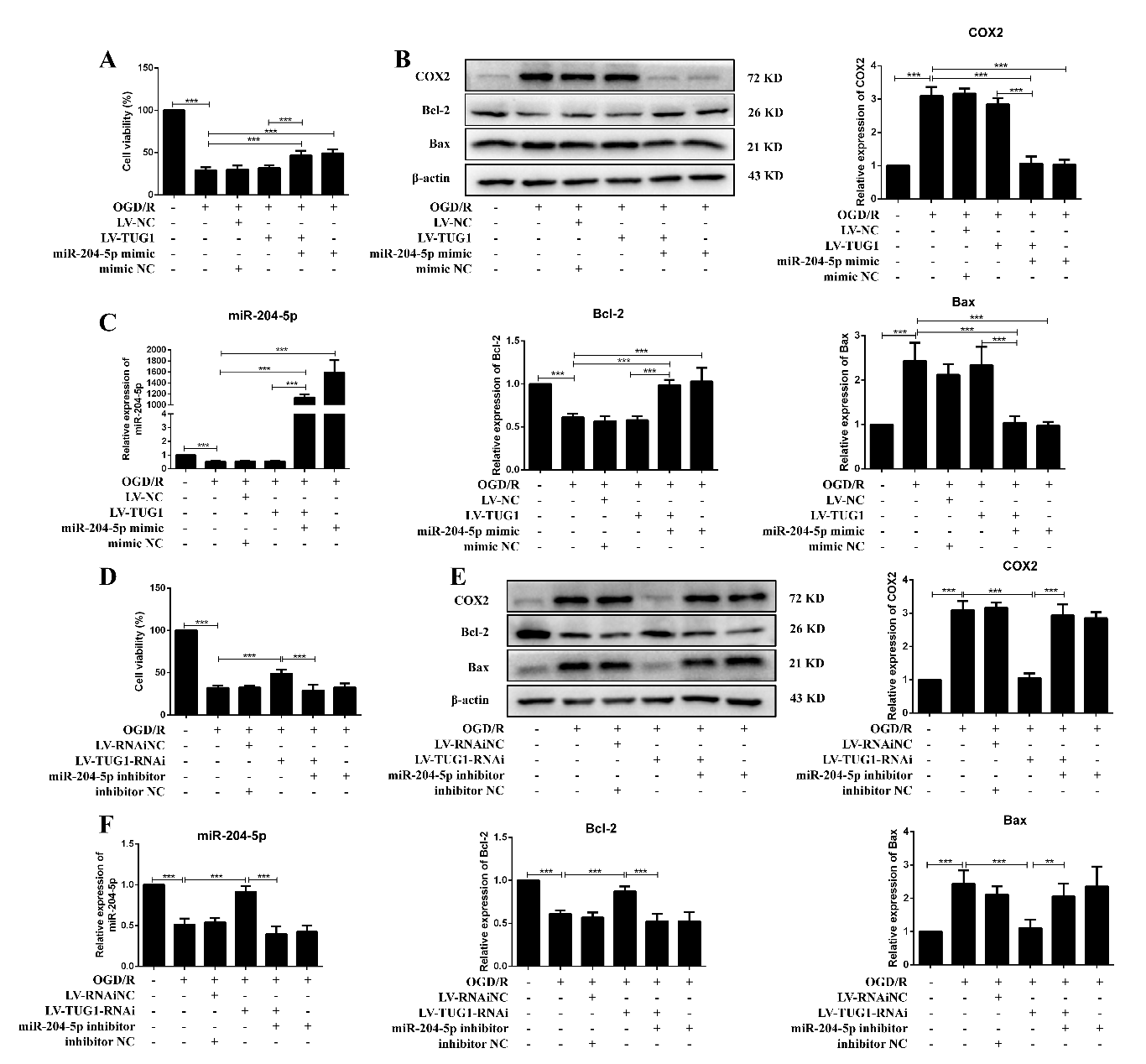


Figure S5 TUG1 knockdown inhibited apoptosis by up-regulating miR-204-5p. **A** MTT assay. (n = 6) **B** Relative protein levels of COX2, Bcl-2 and Bax in neurons after co-transfected with LV-TUG1 and miR-204-5p mimic. (n = 5) **C** Relative expression of miR-204-5p. (n = 5) **D** MTT assay. (n = 6) **E** Relative protein levels of COX2, Bcl-2 and Bax in neurons after co-transfected with LV-TUG1-RNAi and miR-204-5p inhibitor. (n = 5) **F** Relative expression of miR-204-5p in neurons after co-transfected with LV-TUG1-RNAi and miR-204-5p inhibitor. (n = 5) Data are presented as the mean ± SD. ^**^p<0.01, ^***^p<0.001.


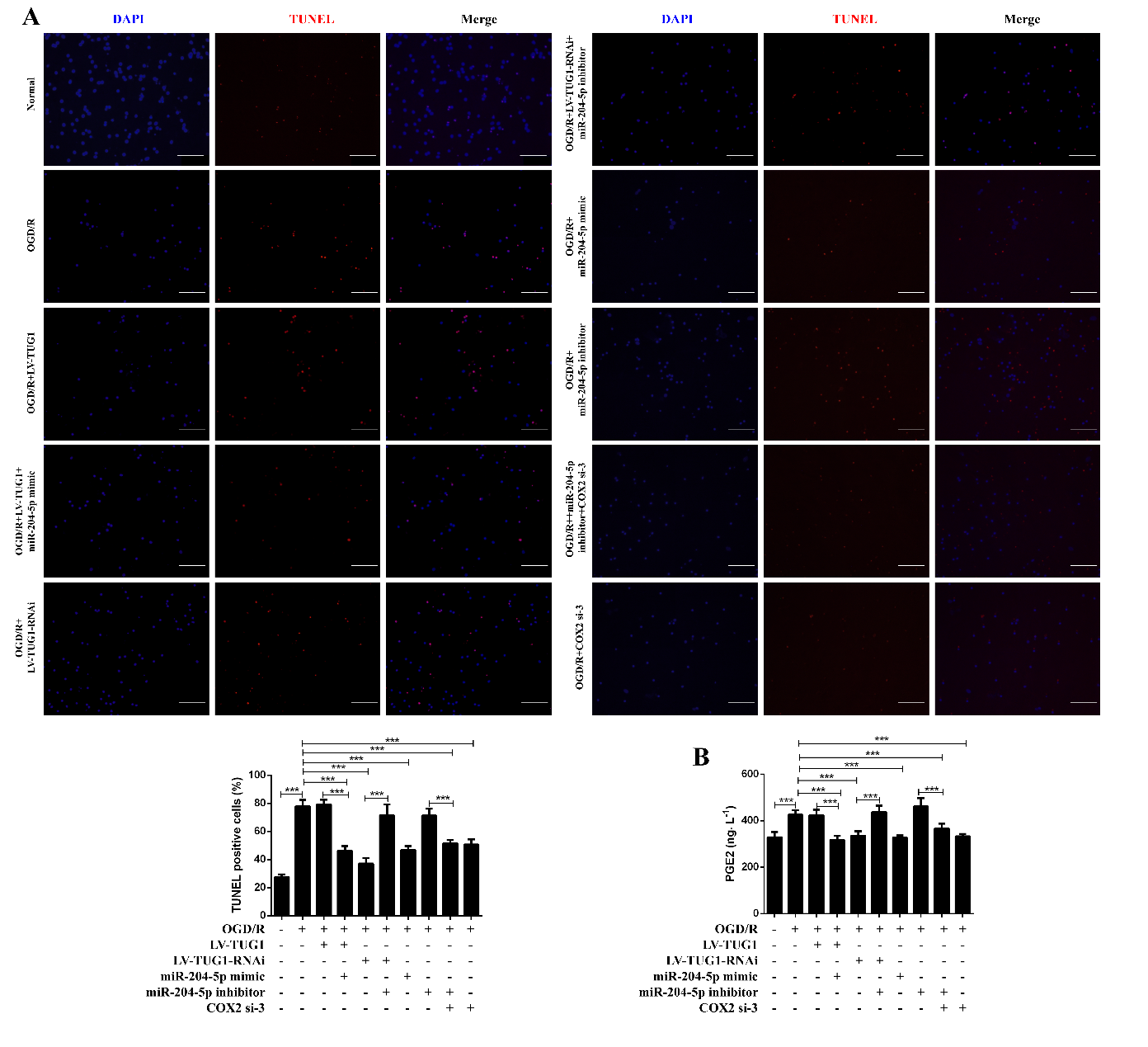


Figure S6 TUG1 and miR-204-5p regulated apoptosis and inflammation in OGD/R neurons. **A** The apoptosis in neurons was detected by TUNEL assay. DAPI (blue), TUNEL (red), 200×, scale bars = 100 μm. **B** The content of PGE2 was detected by ELISIA kits. Data are presented as the mean ± SD (n = 4 in each group). ^***^p<0.001.


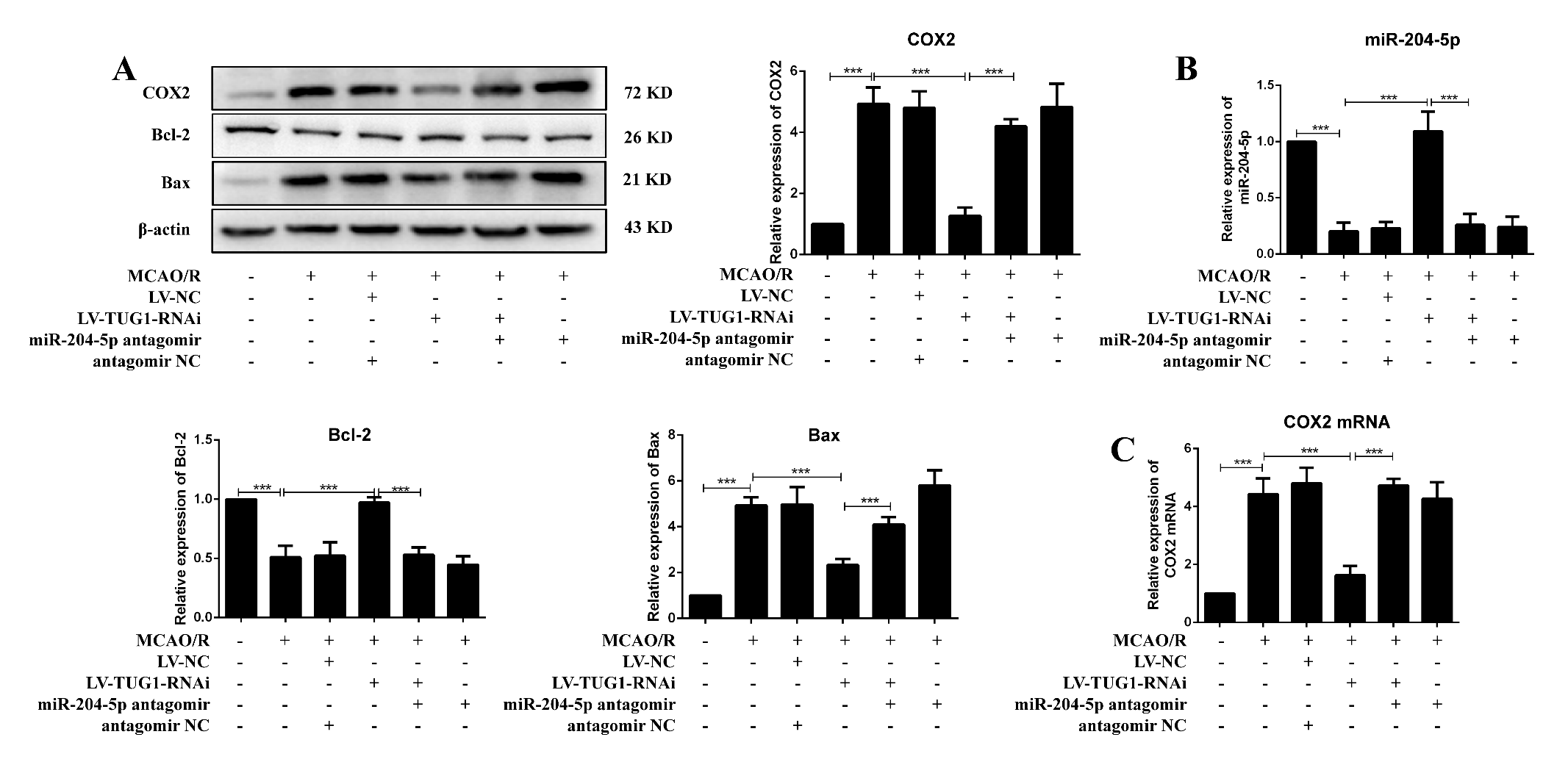


Figure S7 miR-204-5p antagomir reversed the protection effect of TUG1 knockdown in MCAO/R rats. **A** Relative protein levels of COX2, Bcl-2 and Bax in rats after co-infected with LV-TUG1-RNAi and miR-204-5p antagomir. Relative expressions of miR-204-5p (**B**) and COX2 mRNA (**C**) in rats after co-infected with LV-TUG1-RNAi and miR-204-5p antagomir detected by qRT-PCR. Data are presented as the mean ± SD (n = 6 in each group). ^***^p<0.001.
