# Supplementary material for: miR-204-5p is sponged by TUG1 to aggravate neuron damage induced by focal cerebral ischemia and reperfusion injury through upregulating COX2
Source: Cell Death Discov. 2022 Feb 28;8:89. doi: 10.1038/s41420-022-00885-x (PMC8885635; doi:10.1038/s41420-022-00885-x)

**Original WB images**

**Figure 2F Bax**


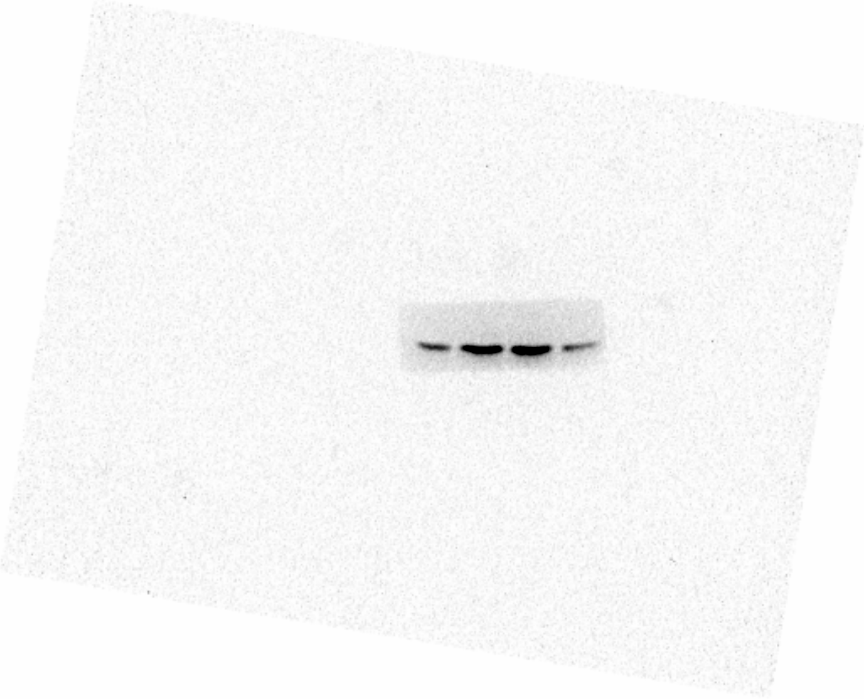


**Figure 2F Bcl-2**


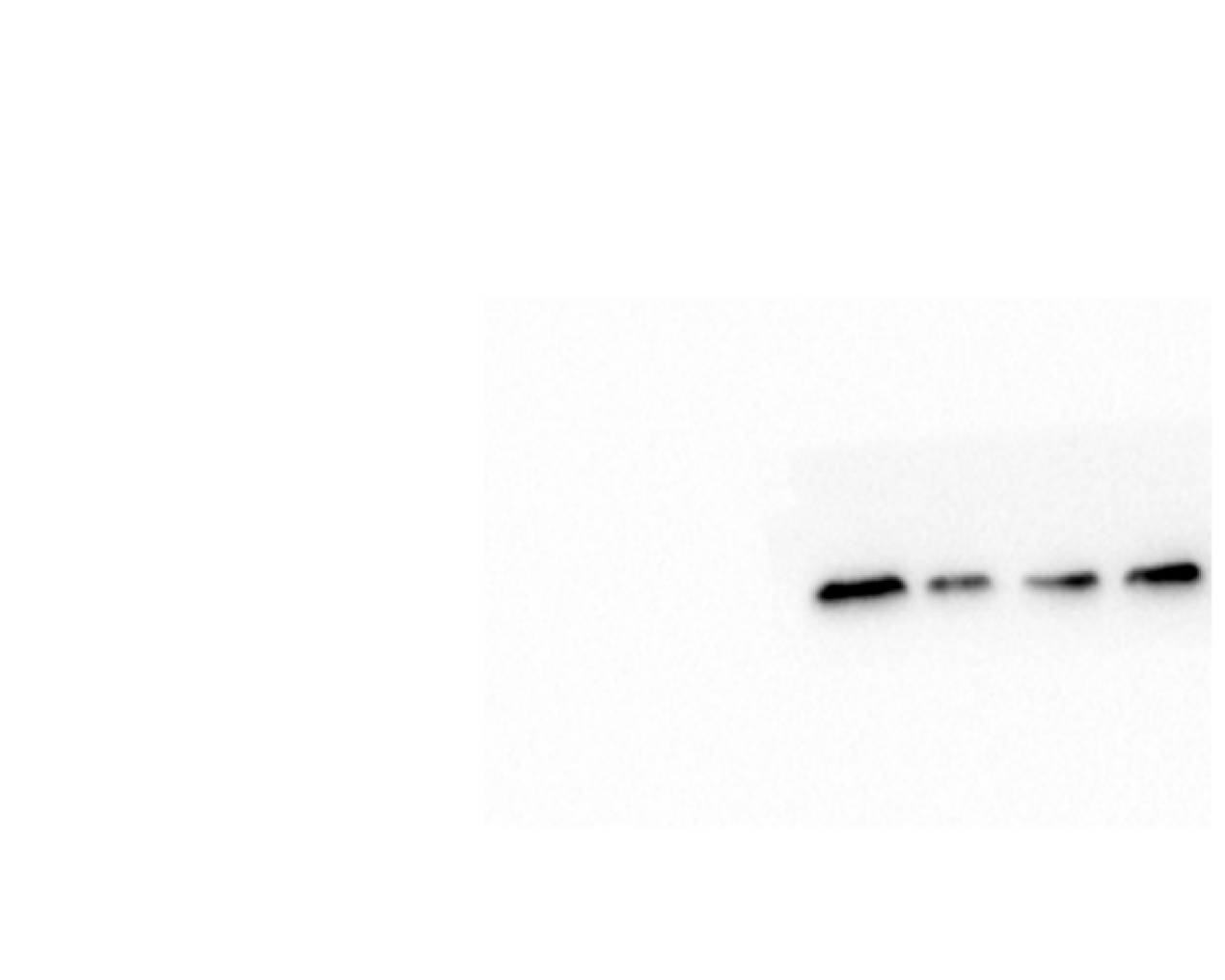


**Figure 2F COX2**


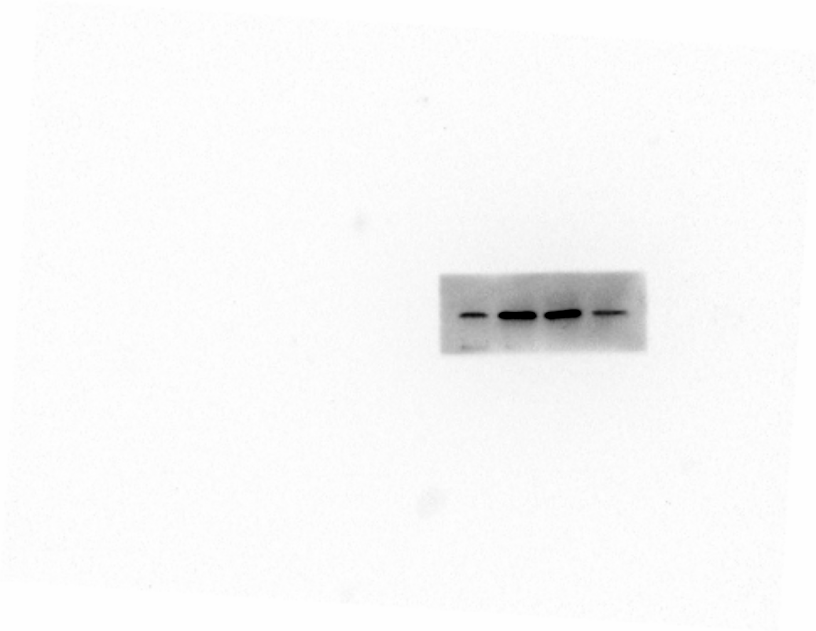


**Figure 2F β-actin**


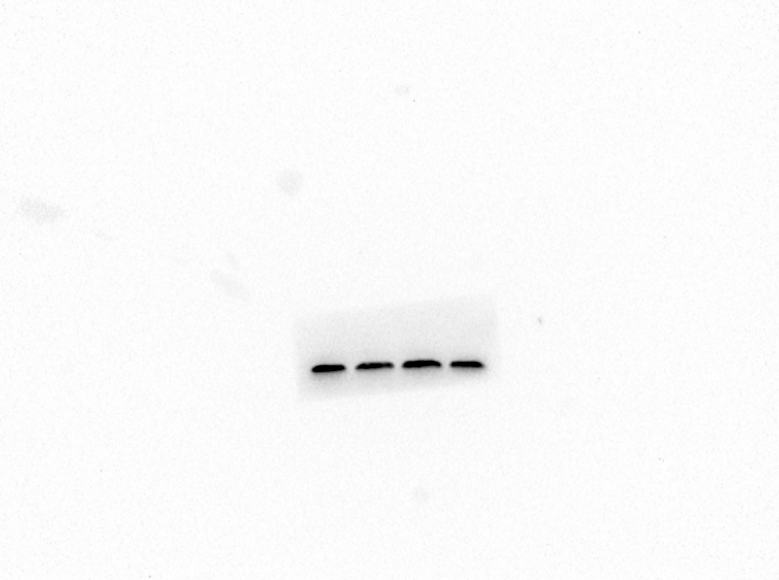


**Figure 3E Bax**


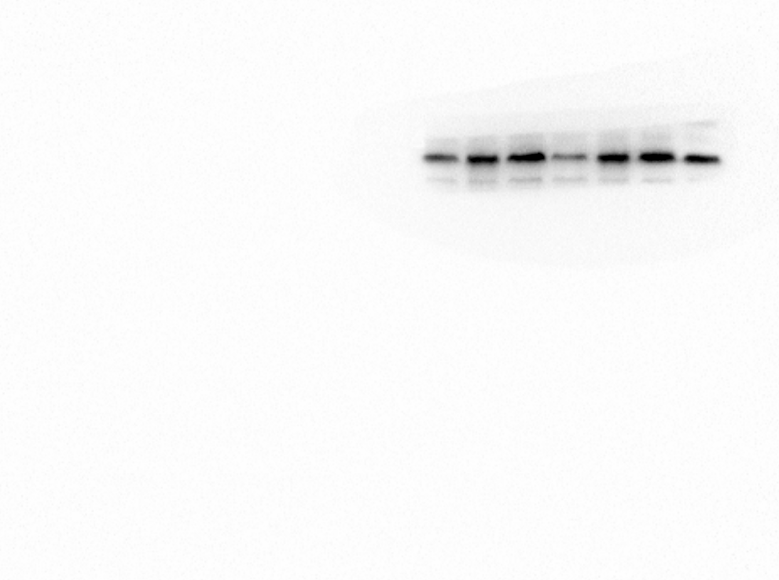


**Figure 3E Bcl-2**


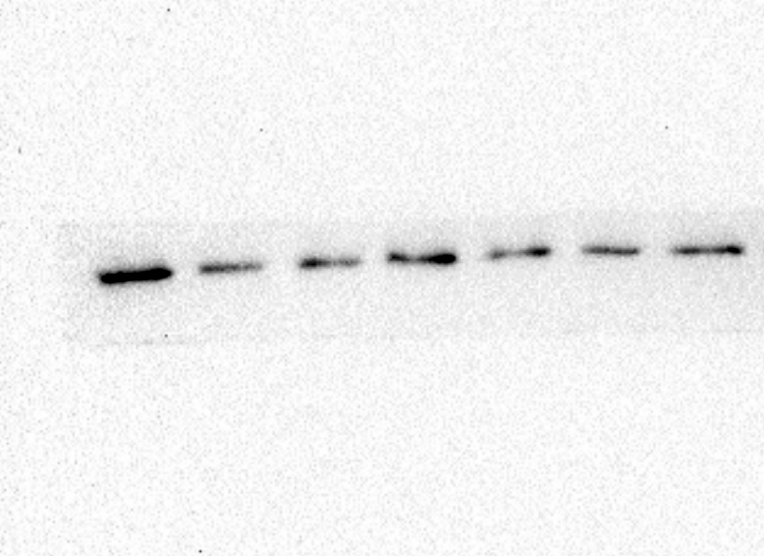


**Figure 3E COX2**


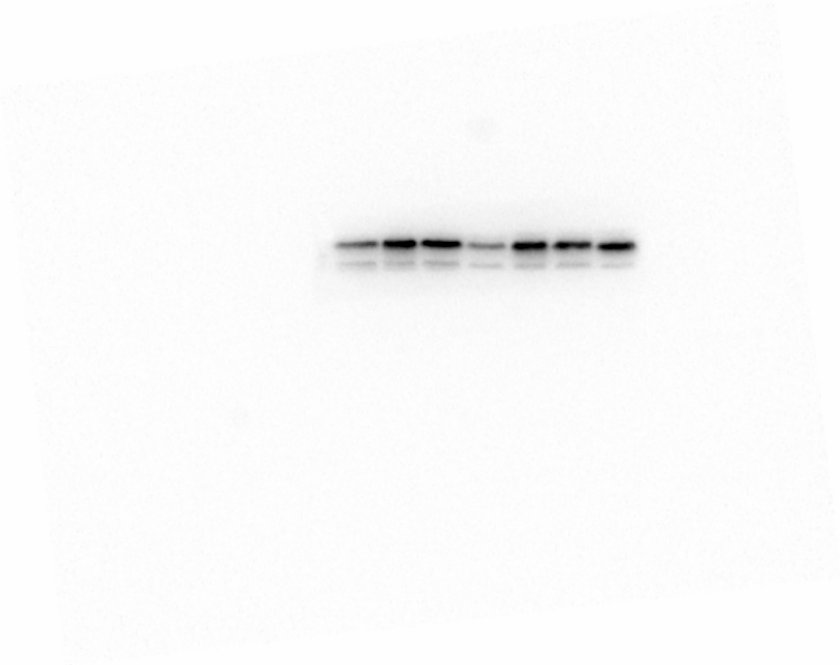


**Figure 3E β-actin**


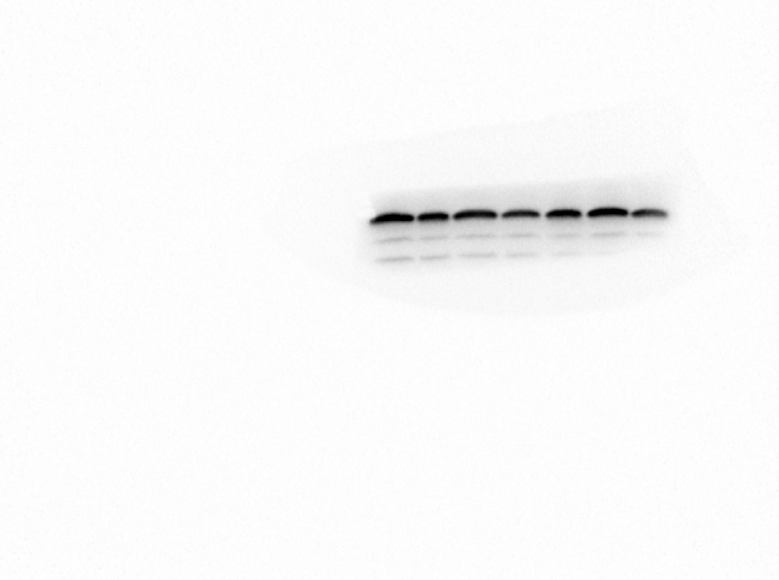


**Figure 4E Bax**


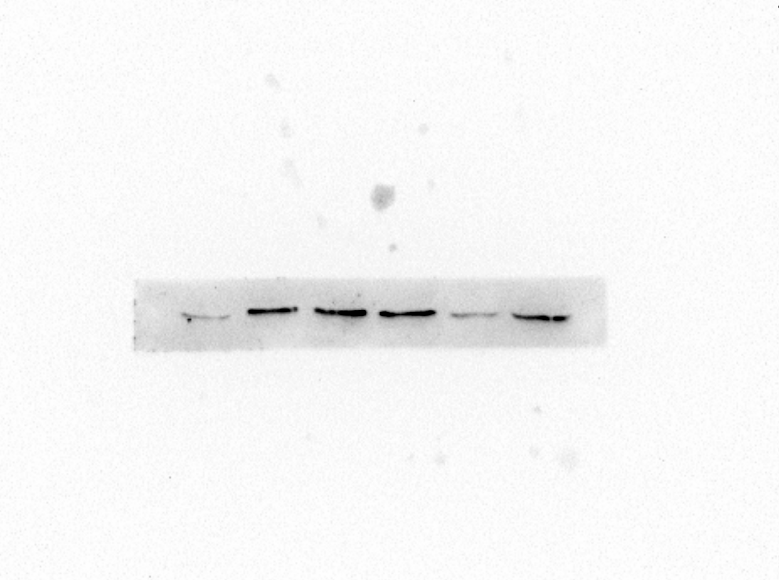


**Figure 4E Bcl-2**


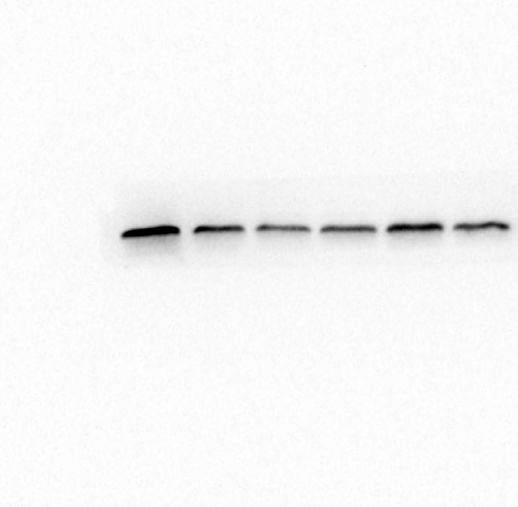


**Figure 4E COX2**


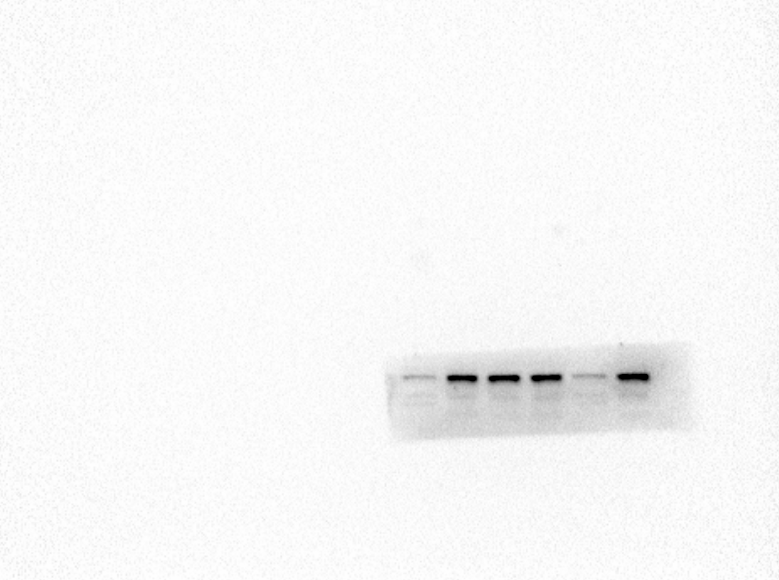


**Figure 4E β-actin**


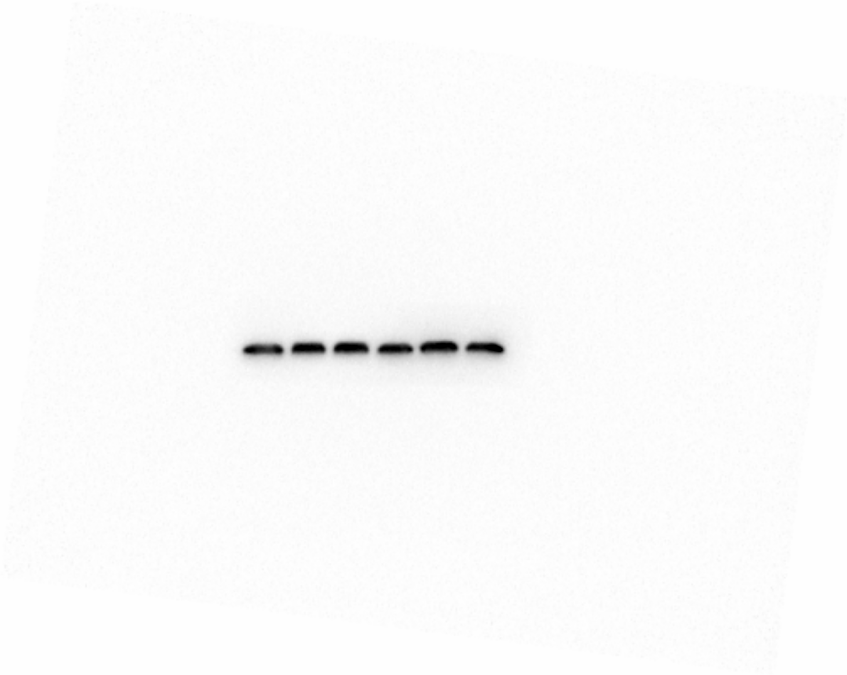


**Figure 5D Bax**


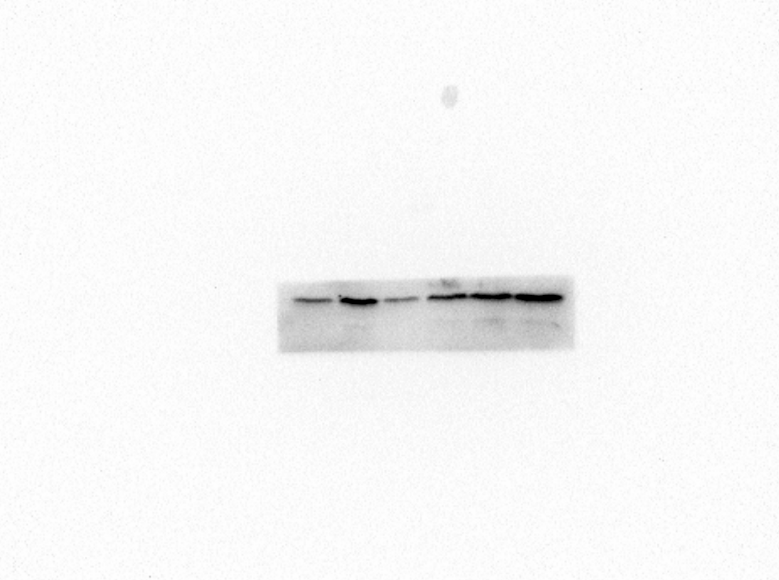


**Figure 5D Bcl-2**


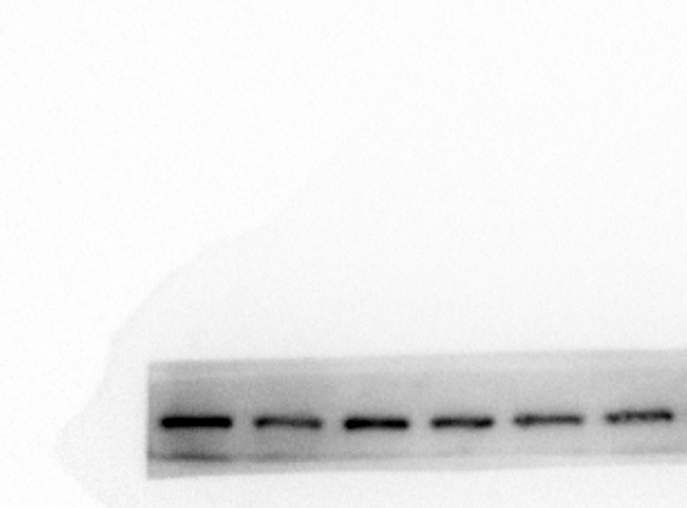


**Figure 5D COX2**


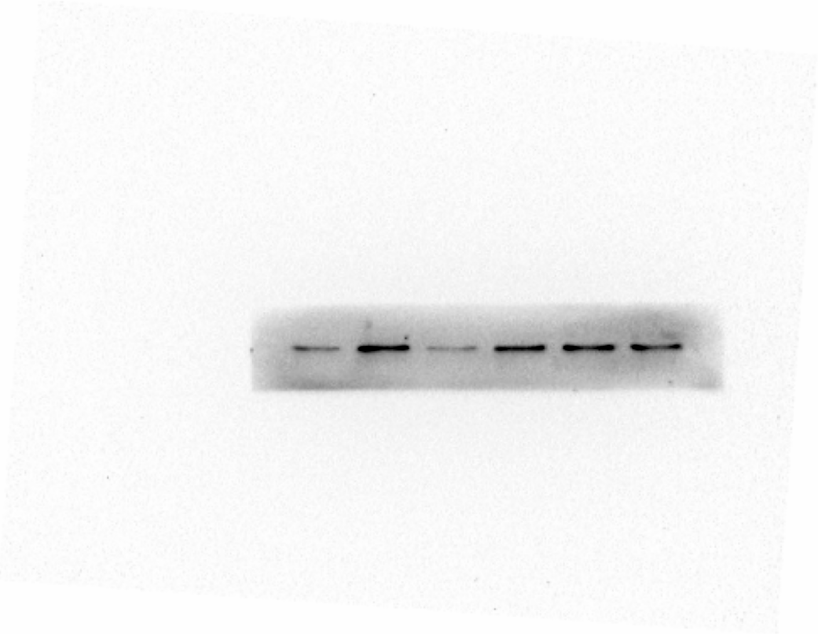


**Figure 5D β-actin**


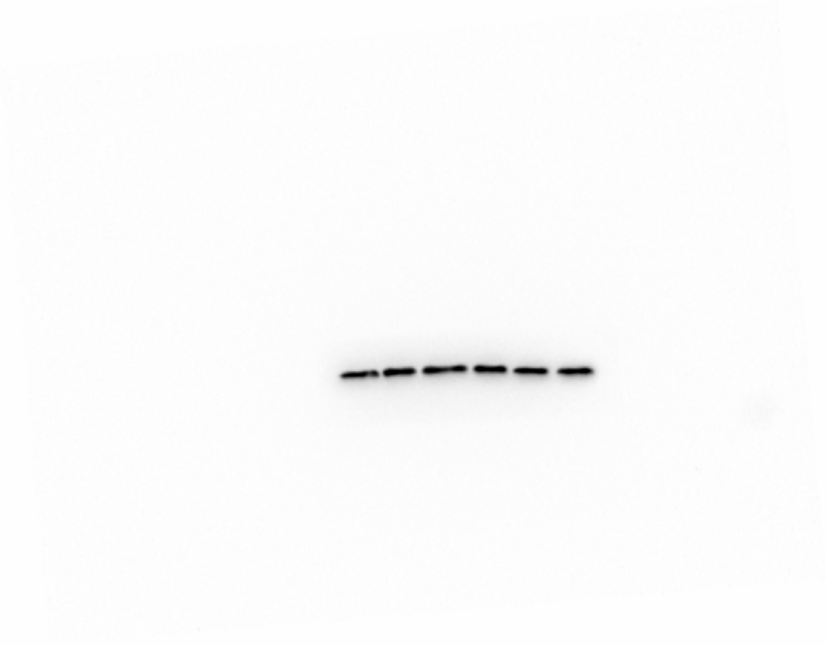


**Figure 6A COX2**


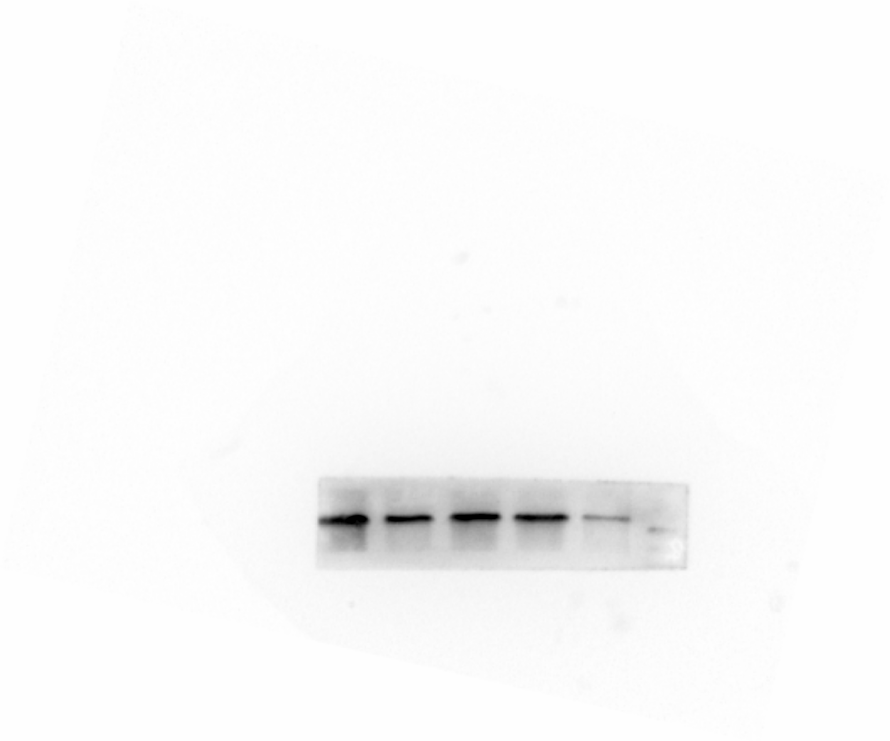


**Figure 6A β-actin**


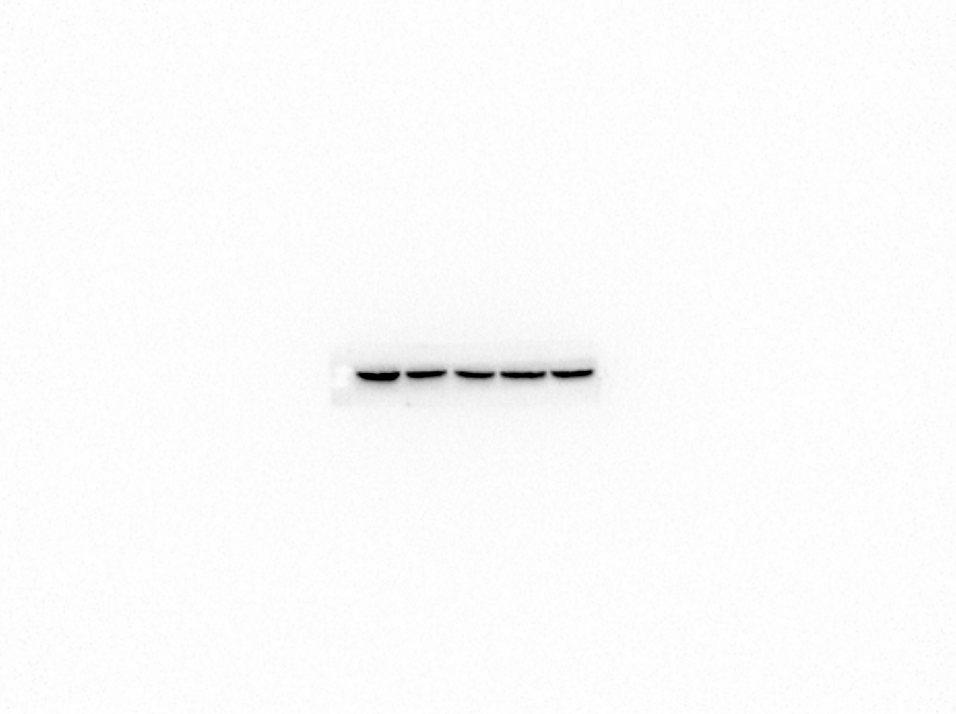


**Figure 6C Bax**


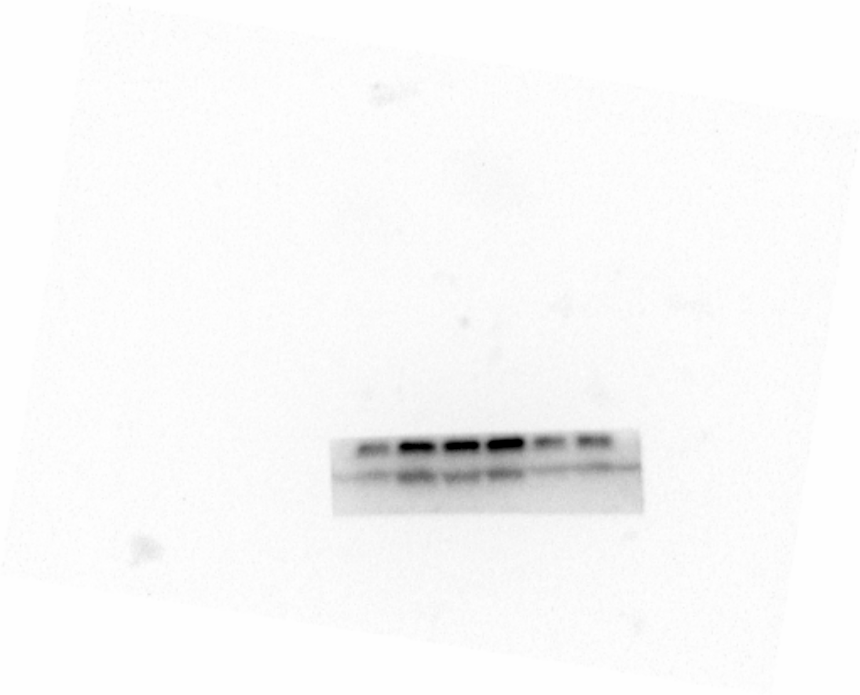


**Figure 6C Bcl-2**


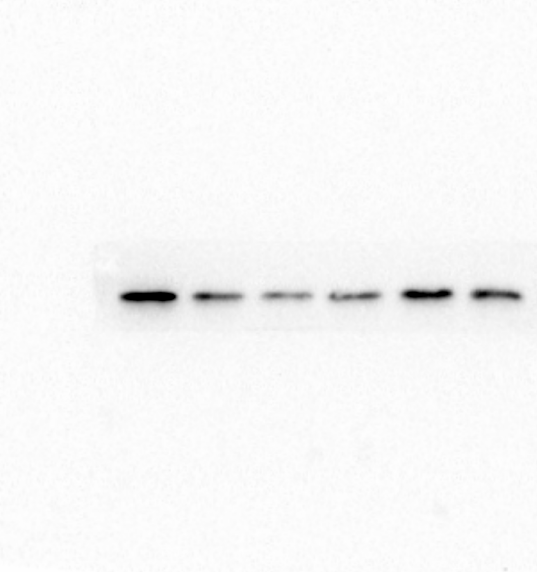


**Figure 6C COX2**


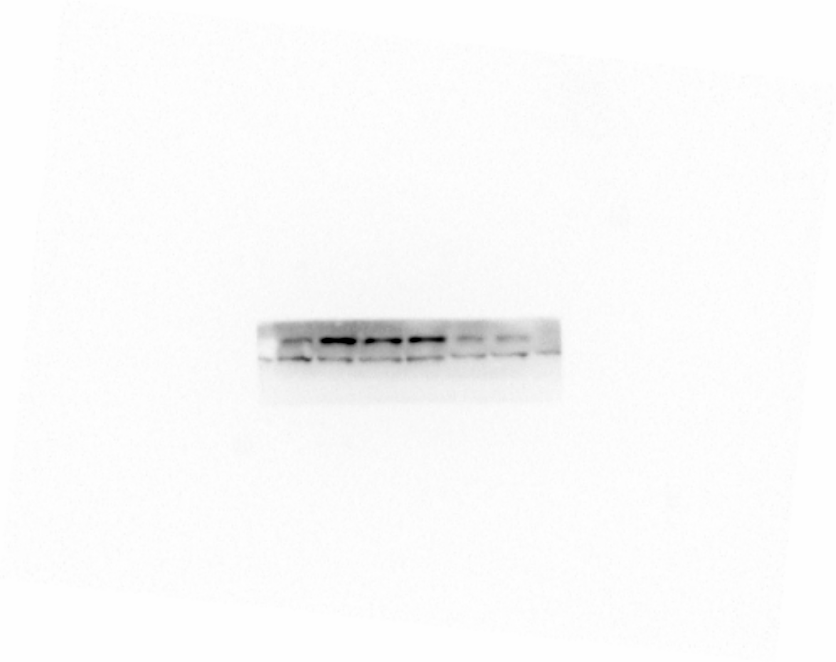


**Figure 6C β-actin**


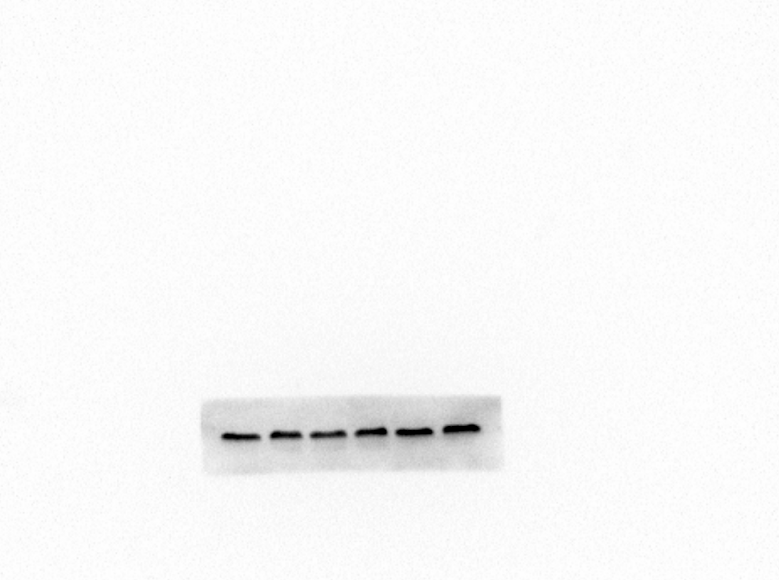


**Figure S5B Bax**


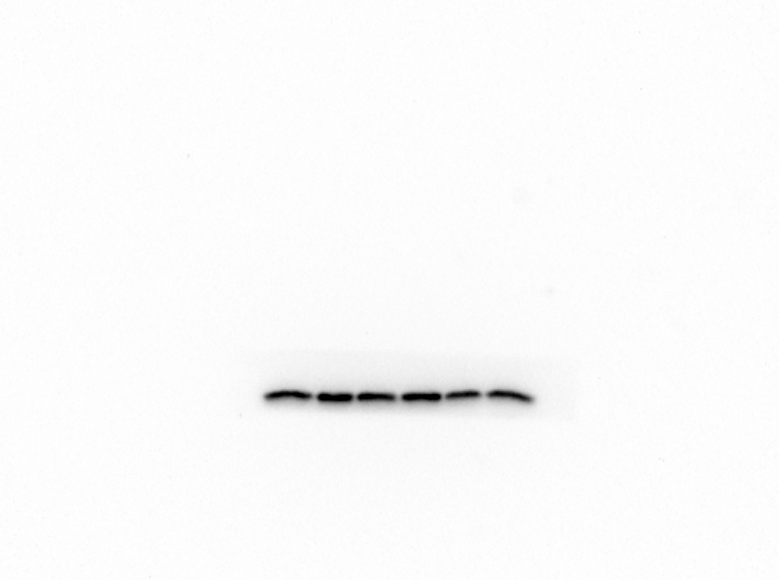


**Figure S5B Bcl-2**


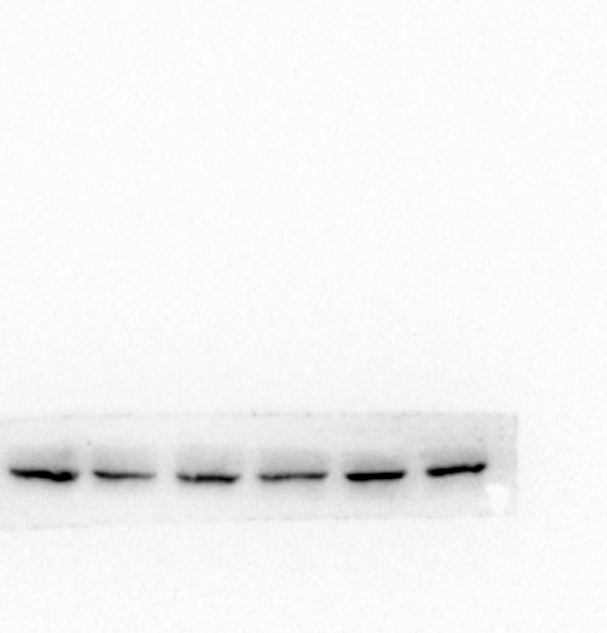


**Figure S5B COX2**


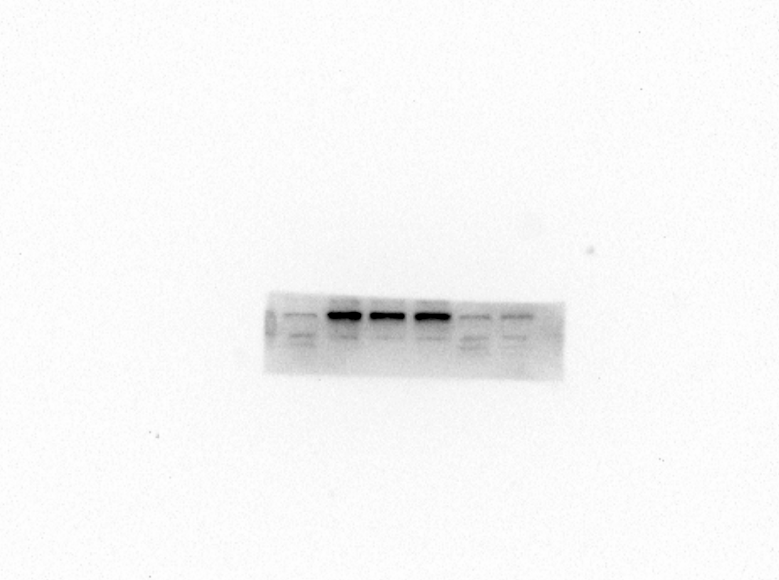


**Figure S5B β-actin**


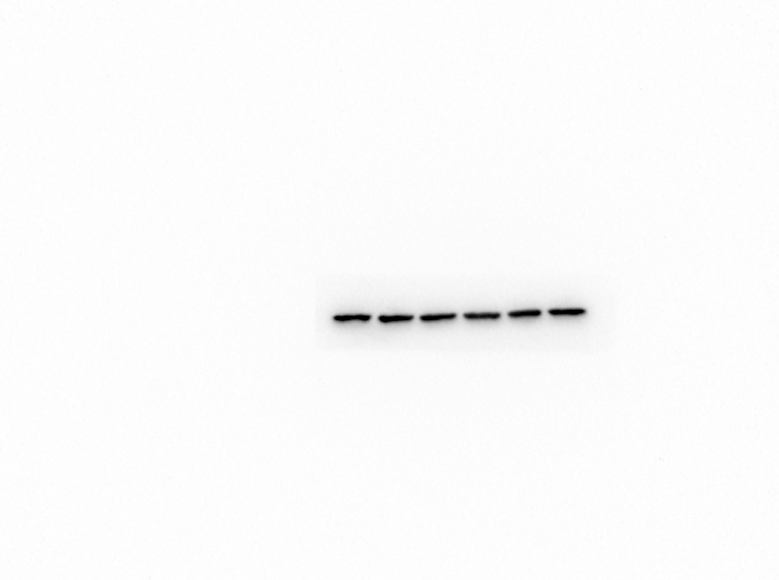


**Figure S5E Bax**


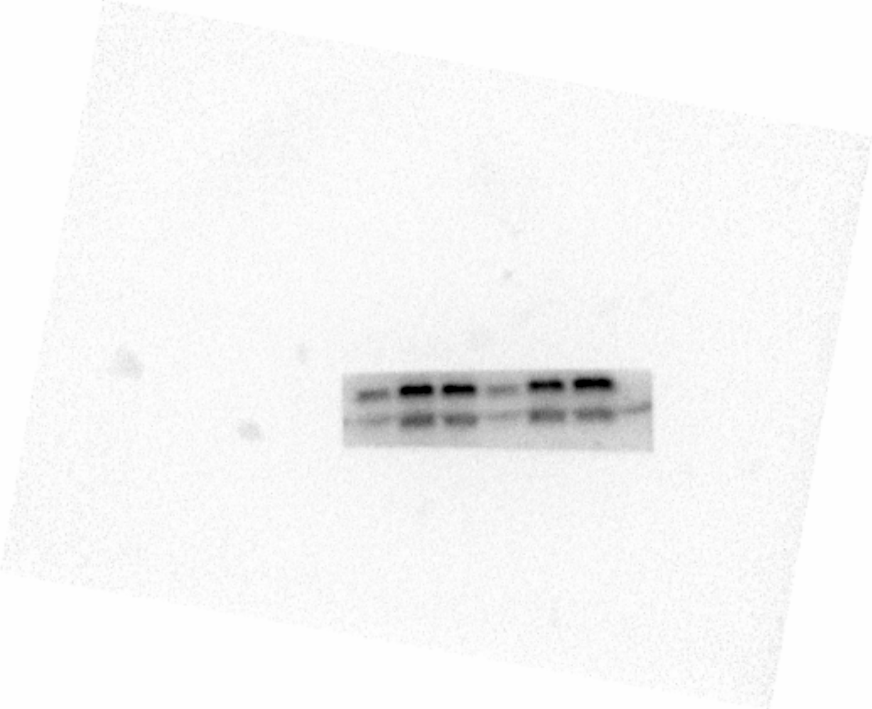


**Figure S5E Bcl-2**


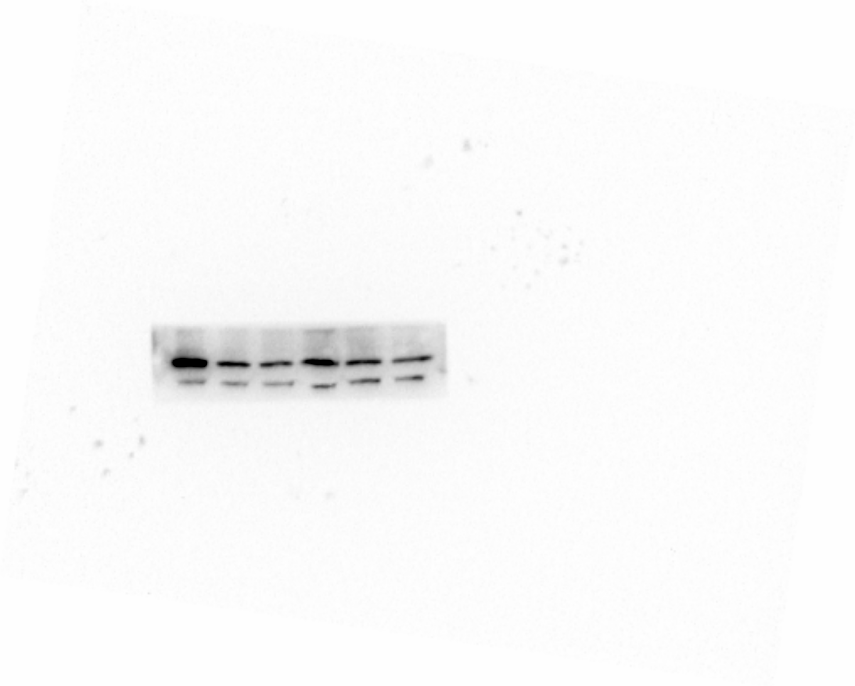


**Figure S5E COX2**


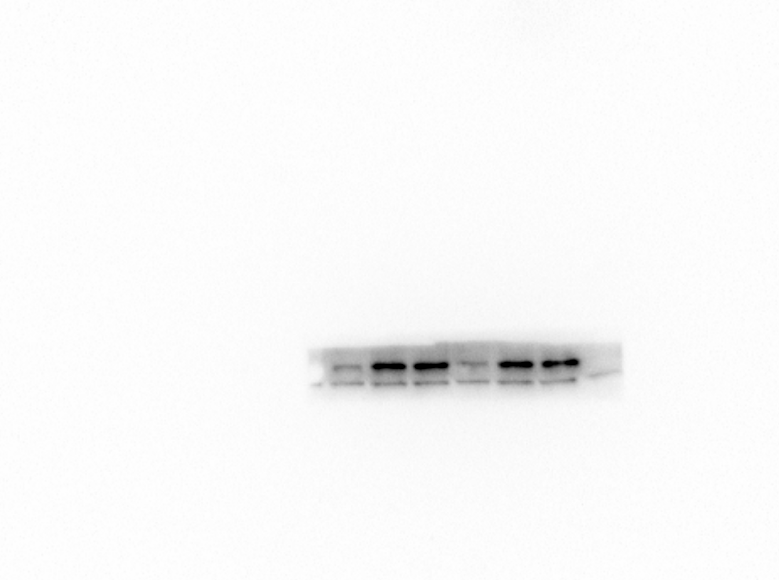


**Figure S5E β-actin**


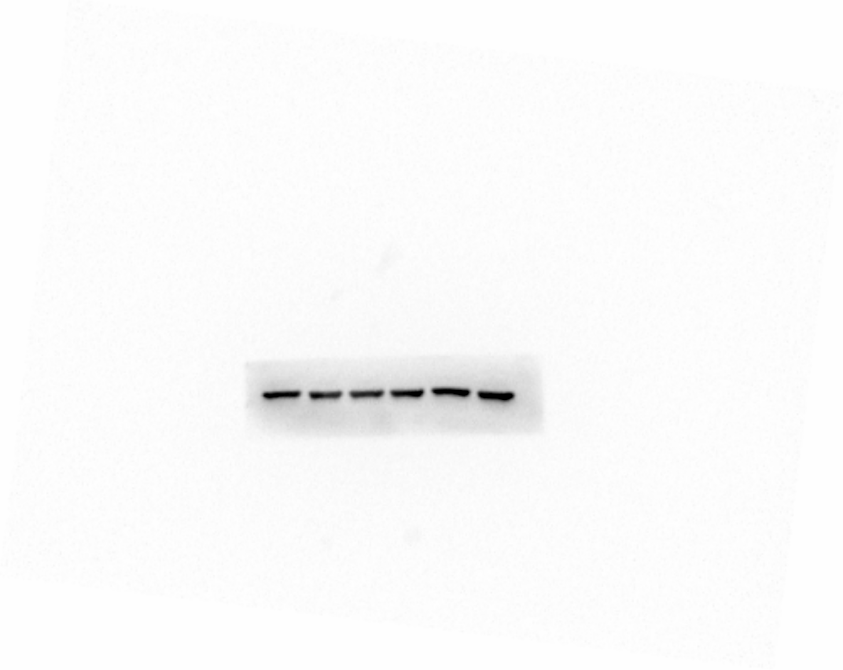


**Figure S7 Bax**


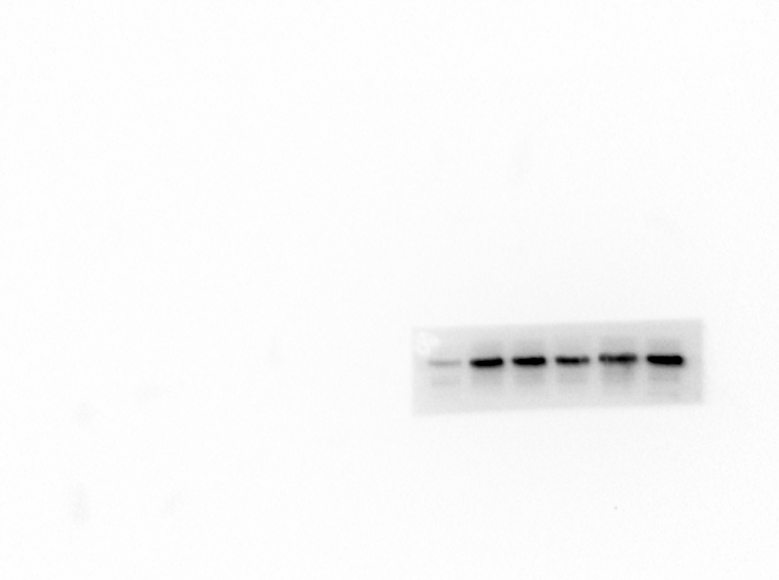


**Figure S7 Bcl-2**


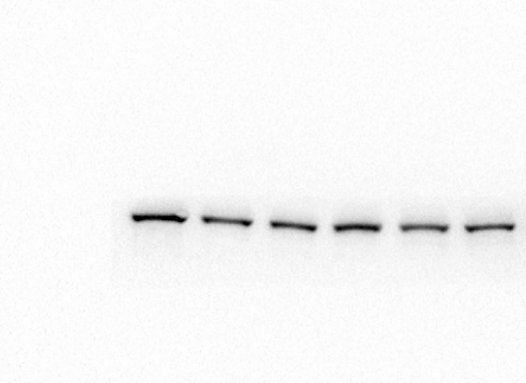


**Figure S7 COX2**


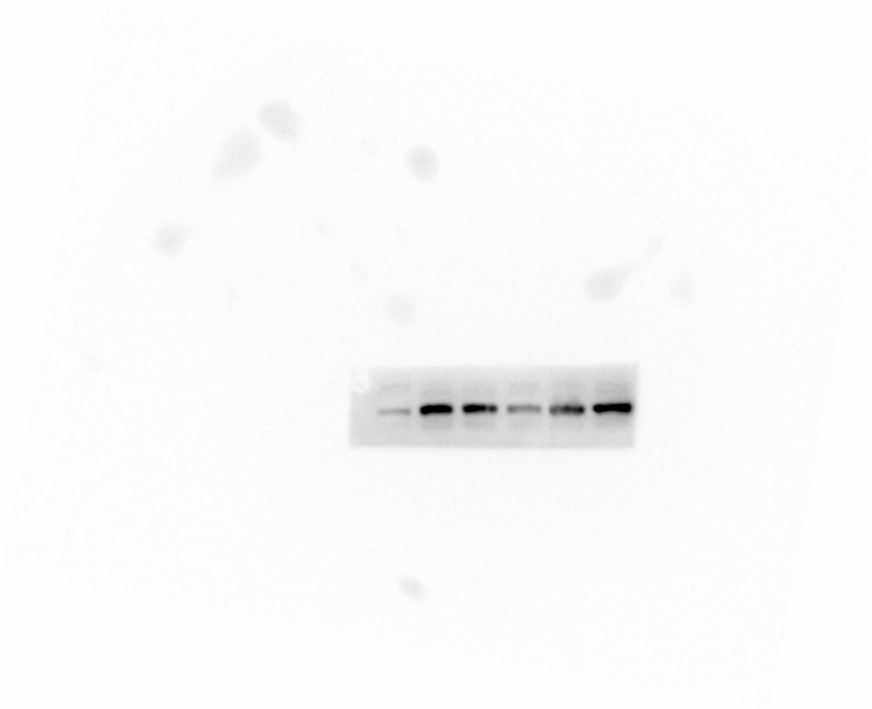


**Figure S7 β-actin**


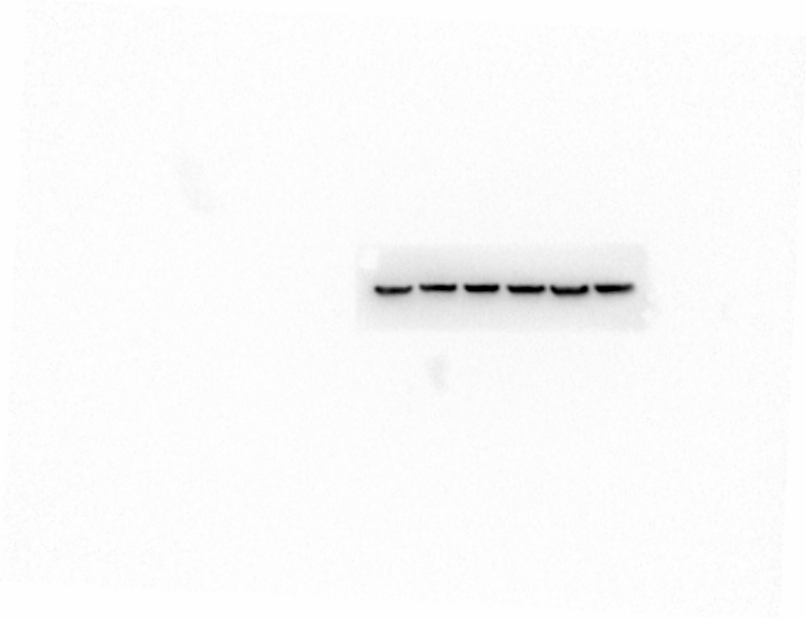

Supplement: Supplementary file 2 — Original WB images [file 41420_2022_885_MOESM2_ESM.docx]
